# Contrast by electron microscopy in thick biological specimens

**DOI:** 10.1111/jmi.70026

**Published:** 2025-08-26

**Authors:** Peter Rez, Lothar Houben, Shahar Seifer, Michael Elbaum

**Affiliations:** ^1^ Department of Physics Arizona State University Tempe Arizona USA; ^2^ Chemical Research Support Department Weizmann Institute of Science Rehovot Israel; ^3^ Department of Chemical and Biological Physics Weizmann Institute of Science Rehovot Israel

**Keywords:** cryo‐electron microscopy, EELS, energy loss imaging, Monte Carlo simulation, multislice simulation, STEM, TEM

## Abstract

The contributions of coherent bright‐field phase and incoherent dark‐field amplitude contrast are investigated for thick biological specimens. A model for a T4 phage is constructed and images simulated for both TEM and STEM phase contrast using a multislice code. For TEM, the fraction of the illumination intensity available for phase contrast imaging is limited by the fraction of electrons in the zero loss peak, the plasmon peak, or the Landau distribution peak for very thick specimens. These were measured from electron energy loss spectra recorded from various thicknesses of vitreous ice. The incoherent amplitude contrast is simulated using the Penelope Monte Carlo code. Noise limits the features that can be distinguished under the low‐dose conditions required for cryo‐EM, even for high electron exposures of 100 electrons/Å^2^. Since in STEM post specimen optics are not used to form the image inelastically scattered electrons contribute to the recorded intensity. In principle STEM should have an advantage over TEM not just for incoherent amplitude contrast but also for coherent phase contrast beyond the limit of weak phase. The simulations suggest that it should be possible to image features in the phage embedded in 1 µm of vitreous ice when collection angles are optimised for bright or dark‐field signals, with best contrast achieved for accelerating voltages of about 700 keV.

## 1 INTRODUCTION

Biological structures at all length scales are intrinsically three dimensional. Although electron microscopes can generate images with atomic resolution, our ability to probe biological structures and map them in 3 dimensions using any ionising radiation is limited by radiation damage.^1^ The strategy to spreading the electron exposure over multiple copies of a given structure has been spectacularly successful in protein structure determination. However it is less clear how such a technique could be applied in general to mapping the unique structures of the contents of a cell or organelle, even at lower spatial resolution. Cryo‐microscopy has proved invaluable in preserving native structure, and the reconstruction from multiple projections, that is, tomography, can be used to reconstruct the 3D structure. In cryo‐electron tomography (cryo‐ET), the images of the projected structure are recorded on a pixelated area detector using the projection lens system of the electron microscope.^2^, ^3^, ^4^, ^5^, ^6^ An alternative technique is cryo‐scanning transmission electron tomography (CSTET),^7^, ^8^ whereby a focused probe is rastered over the specimen and a signal integrated over a range of scattering angles is used to form the image.

It is generally considered that the specimens studied by cryo‐TEM are weak phase objects (WPO), where the contrast arises from interference between unscattered electrons and electrons weakly scattered by the specimen and is a small modulation on a constant background. A consideration of the differences in scattering potential for genetic material, protein and vitreous ice shows that this approximation applies properly only for objects up to about 50 nm thick, even for 300 keV electrons.^9^, ^10^ Specimens typically studied by cryo‐ET are therefore mixed phase/amplitude objects and the thicker specimens are more likely amplitude objects. Inelastic scattering is about 3 times more probable than elastic scattering for biological specimens composed of light elements.^11^, ^12^, ^13^ Even under conditions when phase contrast arises from interference between electrons that pass through the effective aperture, there is still the contribution of amplitude contrast from scattering to angles outside this aperture. There is also amplitude contrast for strong phase objects where the weak phase object condition no longer applies.^9^, ^14^

Inelastic scattering by the specimen leads to a defocus spread due to chromatic aberration in the objective lens. To mitigate the resulting degradation of spatial resolution, that is, image blur, it is customary to record energy‐filtered images from the zero‐loss (elastic scattering) peak. For single particle analysis, such zero‐loss energy filtering is especially effective in recovering high resolution information from specimens of intermediate thickness, where the lost intensity can be compensated by collection of a larger dataset.^15^ For tomography, where sample thickness is often a goal supporting 3D contextual information, we consider the selection of different regimes in the energy loss spectrum in order to maximise the measured signal. The drawback to energy selection is that only a fraction of the scattered electrons are being used for image formation, while the remaining fraction still cause radiation damage. Cryo‐scanning transmission electron tomography (CSTET)^7^ does not suffer these disadvantages since the scattered electrons used in forming images are not affected by objective lens chromatic aberration. Furthermore, since nearly all scattered electrons can be collected using modern pixelated detectors with high dynamic range, it is possible to select angular ranges that maximise signal to noise or optimise spatial resolution. It is even possible to change these dynamically to take account of increased thickness at higher tilts. The ability to examine thick specimens with STEM made it possible to record and reconstruct tomograms of intact, vitrified *Plasmodium* cells,^16^ as well to image cancer cells in liquid phase^17^ and cells of *D. radiodurans* in water when encapsulated by graphene membranes.^18^ Recently, there have also been some developments exploring the possibility of using MeV STEM for nm scale imaging complete cells.^19^

It is also possible to generate phase contrast images by STEM. The most comprehensive method is to record complete diffraction data for each probe position (4D STEM).^20^, ^21^, ^22^ By reciprocity this can be equivalent to TEM bright field with tilted beam illumination covering a range of angles defined by the detector and the camera length. A parallax correction is made for the image shift from each tilt direction and the image contributions from each detector pixel (or range of pixels) are summed. For objects that are not weak phase objects, this results in phase contrast images with much improved signal to noise compared to conventional TEM imaging. This is because all bright‐field electrons are being used in image formation, not just those in a narrow energy range selected by the energy filter, and the effective contrast transfer function transfers a wider range of spatial frequencies.^23^

One of the applications of either cryo‐ET or cryo‐STET is investigating the organisation of genetic material in viral capsids. To explore the contrast that might be achieved we used a model for a T4 phage in various thicknesses of vitreous ice. The model is constructed using the Penelope geometry engine^24^ with a protein capsid and DNA organised in concentric cylinders. This geometry engine can be used to specify the phase grating for the different slices that make up the specimen from an effective potential and an absorption coefficient that represents scattering outside the illumination aperture. The TEM contrast taking account of the need for energy filtering was calculated using a multislice code^25^; the STEM contrast was calculated with the same multislice code summing over contributions from different detector pixels. The simulated STEM images had higher signal to noise since no electrons were lost to inelastic scattering. For purely incoherent imaging the Penelope Monte Carlo code^24^ was used to calculate STEM images for various detector configurations.^26^ The genetic material and capsid were discernible in simulated STEM images of 200 keV electrons in two very different orthogonal projections when the phage was embedded in 400 nm of vitreous ice.

### 1.1 Calculating phase contrast

On average, water makes up about 70% of the content of a cell. Phase contrast comes from the difference in phase shift of a transmitted wave between any macromolecule and water, which in cryo‐microscopy takes the form of vitreous ice.^10^ Proteins are the most abundant macromolecule, followed by genetic material, either RNA or DNA, carbohydrates, and lipids. The phase shift *ϕ* from passing through a material of thickness *t* is

(1) $$\phi =\sigma \tilde{V}t,$$

where $\tilde{V}$ is an average scattering potential (in volts) and *σ* is an interaction constant given by

(2) $$\sigma =\frac{\gamma me^{2}}{\hbar^{2}k}\;,$$

where *m* is the electron mass, *γ* is the Lorentz factor, and *k* is the electron wavevector.

As we are not concerned here with atomic‐resolution structures it is acceptable to use a mean inner potential. For small angles, it is convenient to calculate the phase shift in terms of electron scattering factors:

(3) $$\phi =\frac{\lambda \rho \gamma \sum_{j}f_{j}^{el}\left(0\right)n_{j}}{1.66\sum_{j}n_{j}A_{j}}\;t,$$

where *λ* is the electron wavelength in Å, *γ* is the relativistic Lorentz factor, *ρ* is the density in gm/cc, *n_j_* is the proportion, *A_j_* is the atomic mass, and $f_{j}^{el}\left(0\right)$ is the elastic electron scattering factor for atom *j*. The proportions of the various atoms in vitreous ice, protein and genetic material that were used in the calculations are given in Table 1.

**TABLE 1:** Composition of vitreous ice, protein and genetic material with density used in the calculations.

	Vitreous Ice	Protein	DNA/RNA
C	0	0.31	0.24
N	0	0.08	0.09
O	0.3333	0.08	0.14
H	0.6667	0.525	0.51
S	0	0.005	0
P	0	0	0.02
Density	0.9	1.3	1.7

**FIGURE 1:**
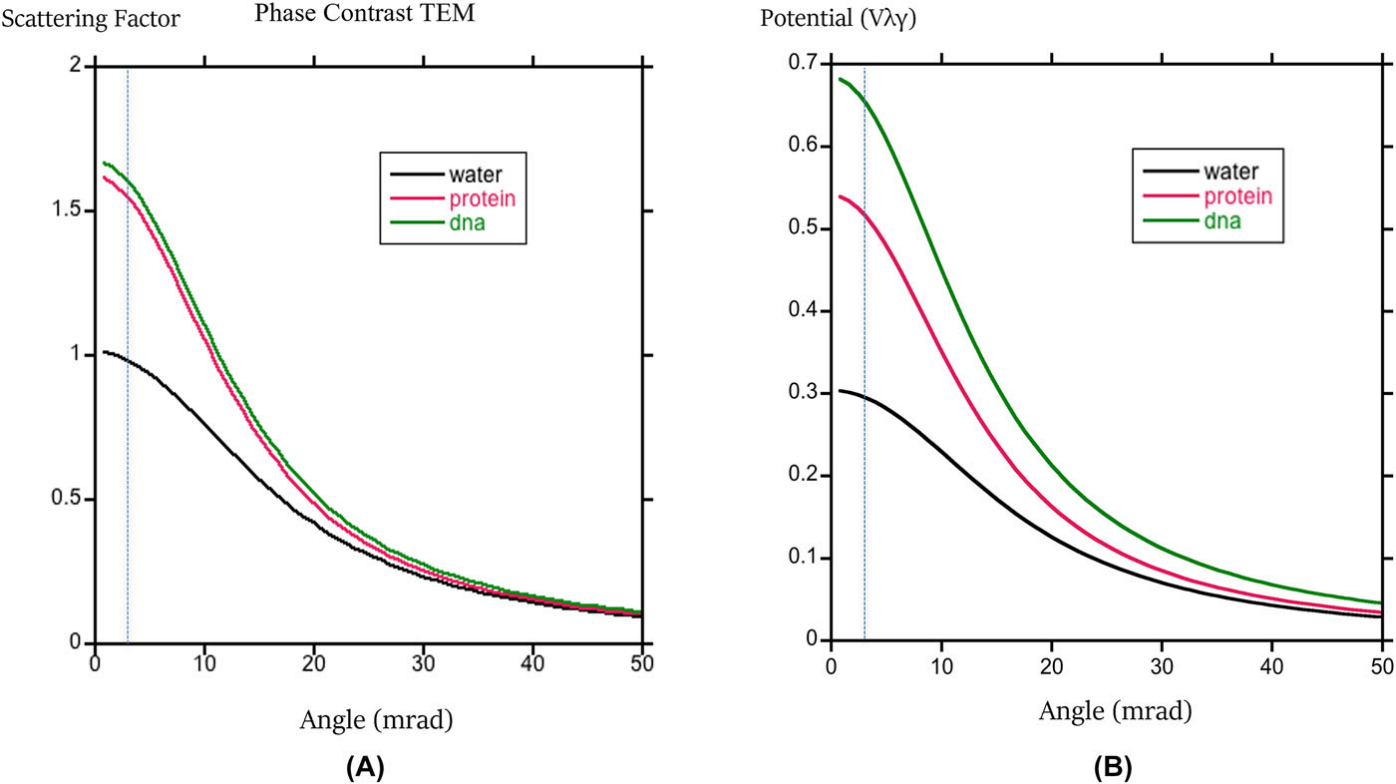
(A) Average electron scattering factor for water, protein and DNA for 200 keV electrons. (B) Contribution to potential (multiplied by γλ) that includes differences in density. The vertical line corresponds to 3 mrad.

Equation (3) shows that the contrast, or difference in phase shift, has two contributions. One is the average electron scattering factor; the other is the difference in density. Figure 1 shows the average electron factor as a function of scattering angle for 200 keV electrons. There is very little difference between protein and genetic material. This is because they have approximately the same ratio of C and O to hydrogen, so any contrast must come from the difference in density. Taking account of the density (Figure 1B) changes the number of atoms per unit volume, and allows for discrimination between protein and DNA and makes it easier to distinguish either protein or DNA from water.

Also note how minimal the change is for angles up to 3 mrad, which corresponds to a resolution of about 0.8 nm, justifying our use of the low angle ($\frac{\sin\theta }{\lambda }=0$) scattering factors.

Considering that most of the structural contrast will be from the difference between protein and vitreous ice we can use Equation (3) to calculate the thickness of protein surrounded by vitreous ice that would give a 1 radian phase difference. The results are shown in Table 2 for different accelerating voltages.

**TABLE 2:** Thickness of protein that gives a 1 radian phase difference from vitreous ice for different accelerating voltages.

Voltage	Thickness
200 keV	47 nm
300 keV	52 nm
1 MeV	64 nm
4 MeV	67 nm

**FIGURE 2:**
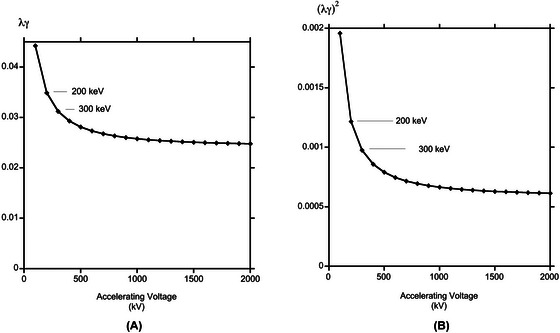
Variation as a function of accelerating voltage of (A) electron scattering factor or Fourier coefficient of potential, λγ, and (B) mean free path for incoherent scattering ${\left(\lambda \gamma \right)}^{2}$.

Note that using 1 MeV or 4MeV electrons makes very little difference. This is because the product of the Lorentz factor *γ* used in the relativistic correction for the electron mass and the relativistic expression for the wavelength, *λ*, in Equation (3) for the electron scattering potential does not change much with accelerating voltage as shown in Figure 2A, graphically illustrating that very high accelerating voltages do not greatly extend the range of validity of the weak phase object approximation. The elastic or inelastic mean free path from incoherent scattering is proportional to ${\left(\lambda \gamma \right)}^{2}$ and its variation with accelerating voltage is shown as Figure 2B. Since the limit to the thickness of material that can be examined is related to the number of scattering events, or mean free path, it would appear that MeV accelerating voltages brings no advantage.

Setting the phase‐difference limit for a WPO as 1 radian is already rather generous. From an expansion of sin*θ* the difference from the small angle approximation, * θ*,  would be 16%, which is somewhat problematic for quantitative analysis of data. From these estimates it can be seen that thick biological objects are not weak phase objects.

**FIGURE 3:**
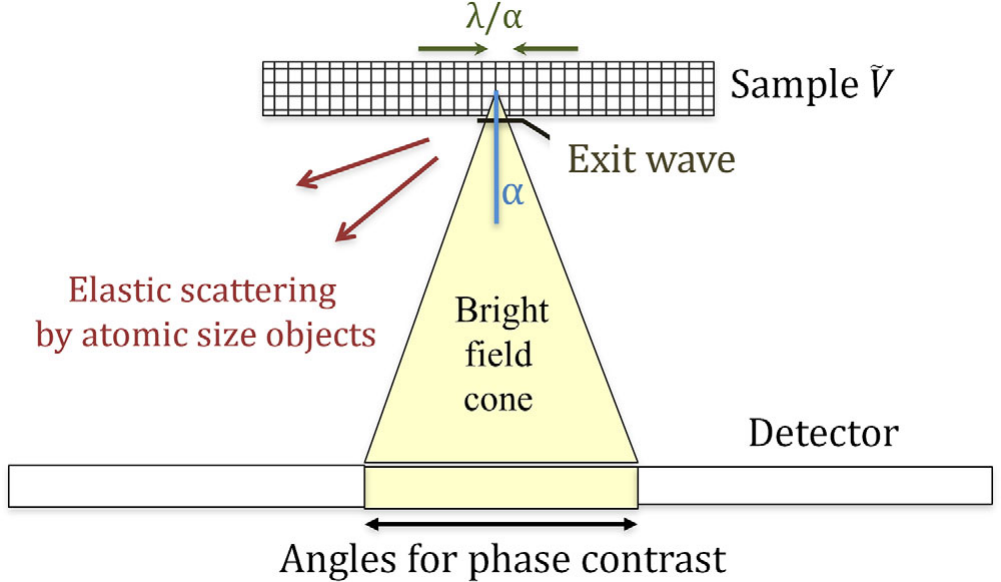
The illumination convergence angle ±*α* is the range of angles used for forming phase contrast images. Phase objects smaller than the resolution *λ*/2*α* are lost and interpreted as amplitude contrast.

Especially for thick samples, there is not only phase contrast but also amplitude contrast that arises from electrons that are scattered outside the angular range used for TEM imaging or STEM illumination shown schematically in Figure 3. We note that for a given spatial resolution *d*, the illumination semi‐convergence must include scattering vectors up to angle $\alpha =\frac{\lambda }{2d}$. Scattering to higher angles will be rendered as amplitude contrast, even from a weak phase object. For thick specimens, it is common to reduce the convergence in order to increase the depth of field, particularly for tomography where simple projections are reconstructed to 3D most conveniently. Nonetheless, significant phase contrast in STEM is observed experimentally up to the limiting resolution defined by the convergence.

The angular range could be limited by the contrast transfer function (CTF) as modified by the appropriate envelope functions for temporal and spatial incoherence. This will depend on the operating conditions of the microscope, in particular the defocus.^27^

**FIGURE 4:**
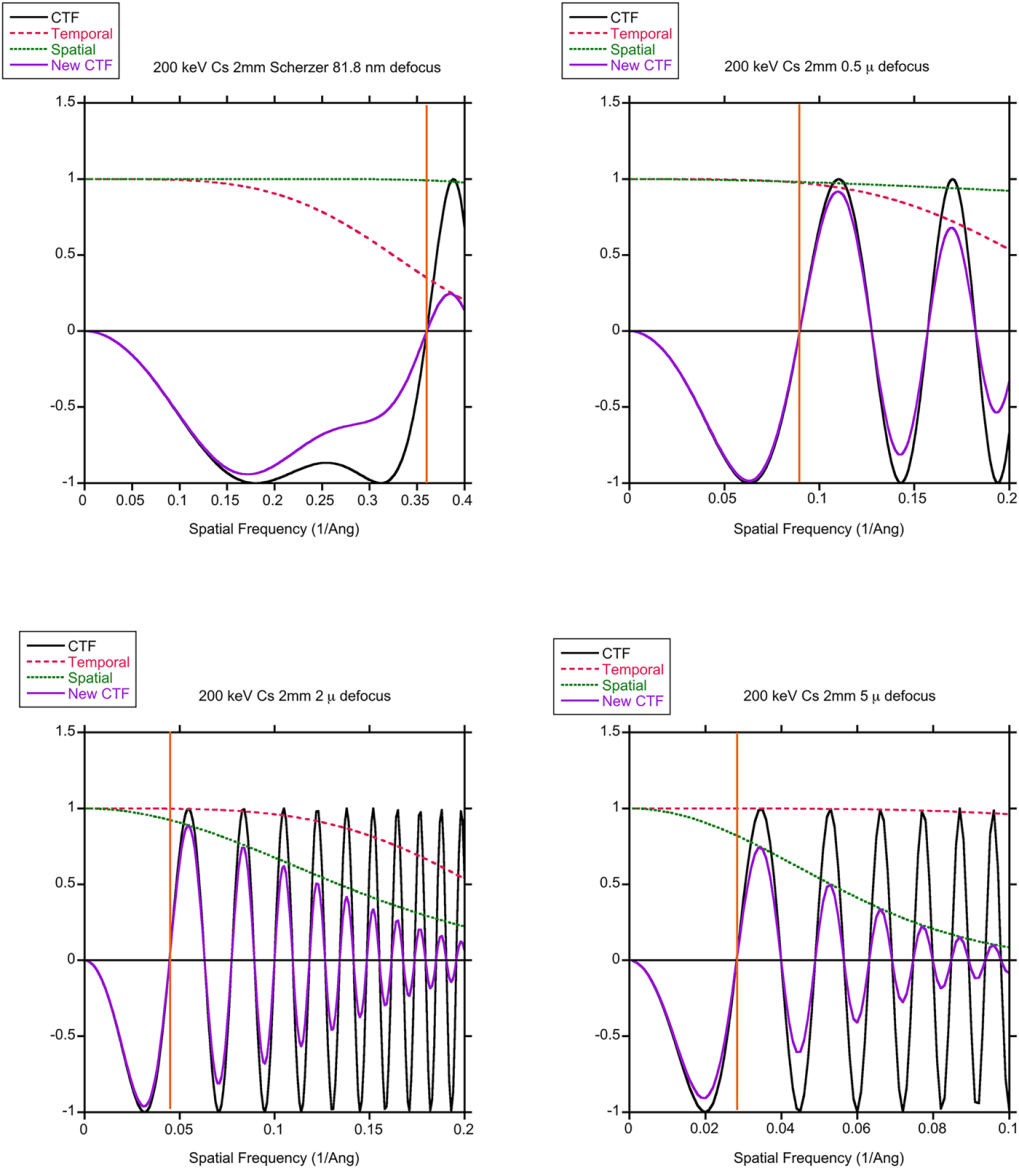
Contrast transfer functions for a FEI F20 200 keV, *C_s_* = 2.0 mm, beam divergence 0.1 mrad, showing spatial frequency range with limit (orange line) for (A) Scherzer focus (81.8 nm), focal spread 10 nm (B) a defocus of 500 nm and low resolution imaging with 2 µm defocus (C) and 5 µm defocus (D) all with focal spread 50 nm.

Representative CTFs and envelope functions are shown in Figure 4 for 200 keV FEI F20 Cs = 2.0 mm for both a high resolution imaging condition close to Scherzer focus, an intermediate focus of 500 nm appropriate for features approximately 1 nm in size and low resolution conditions that might be used to pick out the overall shapes of biomolecules. The form of the contrast transfer function is very similar for a 300 keV Krios, Cs 2.7 mm, as shown in Figure S1. As the defocus increases, the coherent passband shifts to lower spatial frequencies, so that the object becomes easier to see. For higher spatial frequencies (requiring high collection angles), there are oscillations in the transfer function; the resulting contrast inversions must be corrected in the image for proper interpretation. These oscillations are damped by the temporal coherence function due to the spread of energies from the electron gun and instabilities in the objective lens power supply, as well as the spatial coherence function that arises from the incident beam convergence.^28^ For STEM, the equivalent of the beam convergence is the angle subtended by a detector pixel. The temporal coherence is more important near Scherzer, or optimum defocus for high‐resolution imaging, whereas the spatial coherence is more significant at large values of defocus. These act to impose an effective cut off in scattering angles. Alternatively, the angular range could be defined by a phase plate acting on the scattered electrons. In some cases the angular range might be defined by an objective aperture. Without loss of generality we can state that this scattering does not contribute to a bright‐field phase contrast image shown schematically in Figure 3. Its strength can be incorporated into an absorption coefficient *µ*

(4) $$\mu =\frac{\gamma^{2}\rho }{1.66\sum_{j}n_{j}A_{j}}\;\int_{\theta_{min}}^{\theta_{max}}\sum_{j}n_{j}{\left[f_{j}^{el}\right]}^{2}d\Omega ,$$

where *θ_min_* is the angular limit for phase contrast (i.e., the highest spatial frequency cut off given by the illumination convergence in STEM) and *θ_max_* is a maximum scattering angle. For application to STEM dark‐field imaging this could be the maximum angle subtended by an annular detector. The absorption leads to an attenuation

(5) $$I=\exp\left(-\mu t\right)I_{0}$$

in the bright‐field image. This would be the same in STEM for a small objective aperture defining the bright‐field image.

### 1.2 Multislice calculations

The multislice algorithm^29^ is a standard method for calculation of atomic resolution images in materials science, where one iterates between multiplying the real space wave function by a phase grating exp(iϕ) followed by propagation through free space by a distance Δ*t* to the next slice. This is most conveniently calculated by taking the Fourier transform of the wavefunction and multiplying by a propagator $\exp(\frac{i\theta^{2}}{2\lambda }\Delta t)$, and then performing the inverse Fourier transform.^30^ Here we apply the multislice algorithm to calculate phase contrast images of biological structures in thick specimens at nm resolution.

The absorption from scattering to angles outside the domain of the multislice calculation can be effectively taken into account by making the potential complex, where the imaginary part of the potential is $\frac{\mu }{2}$.

The slice thickness should be chosen to adequately sample changes in the potential from changes in the structure. If the changes are slowly varying the slice thickness will depend on the acceptable error. An analysis of the error *E* for a single slice shows that it varies as the Δ*t* ^2^ where Δ*t* is the slice thickness

(6) $$E=\frac{1}{2}\;\frac{\lambda \rho \gamma \sum_{j}f_{j}^{el}\left(\theta_{M}\right)n_{j}}{1.66\sum_{j}n_{j}A_{j}}\times \frac{\pi \theta_{M}^{2}}{\lambda }\Delta t^{2},$$

where *θ_M_* is the largest angle defined from the sampling in real space in the calculation. If the cell representing the structure has length *L* which is divided into *N* elements the largest angle *θ_M_* is

(7) $$\;\theta_{M}=\frac{\lambda N}{2L}.\;$$

For a 128 nm × 128 nm field of view needed for a T4 phage capsid, the maximum angle in the multislice for 200 keV is 5 mrad along the side of the cell. Since the number of slices for a fixed thickness is inversely proportional to the slice thickness the overall error scales as the slice thickness. The reason why the error in multislice calculations can be quite small in practice is that the scattering factor or potential is falling for large scattering angles, so even when multiplied by *θ* ^2^ it can still be acceptably low. Using this criterion given by Equation (6) for the phage in vitreous ice, a slice thickness of 2 nm would ensure an overall error of less than 10%.

The wavefunction in reciprocal space at the exit surface can then be phase shifted by the aberration function

(8) $$\chi =\frac{\pi }{\lambda }\;\left(\frac{1}{2}C_{s}\theta^{4}-\Delta f\theta^{2}\right)$$

multiplied by the appropriate envelope functions. The Fourier transform of the phase shifted and scattering angle‐limited wavefunction is a complex amplitude from which the final image intensity can be derived as the modulus squared.

**FIGURE 5:**
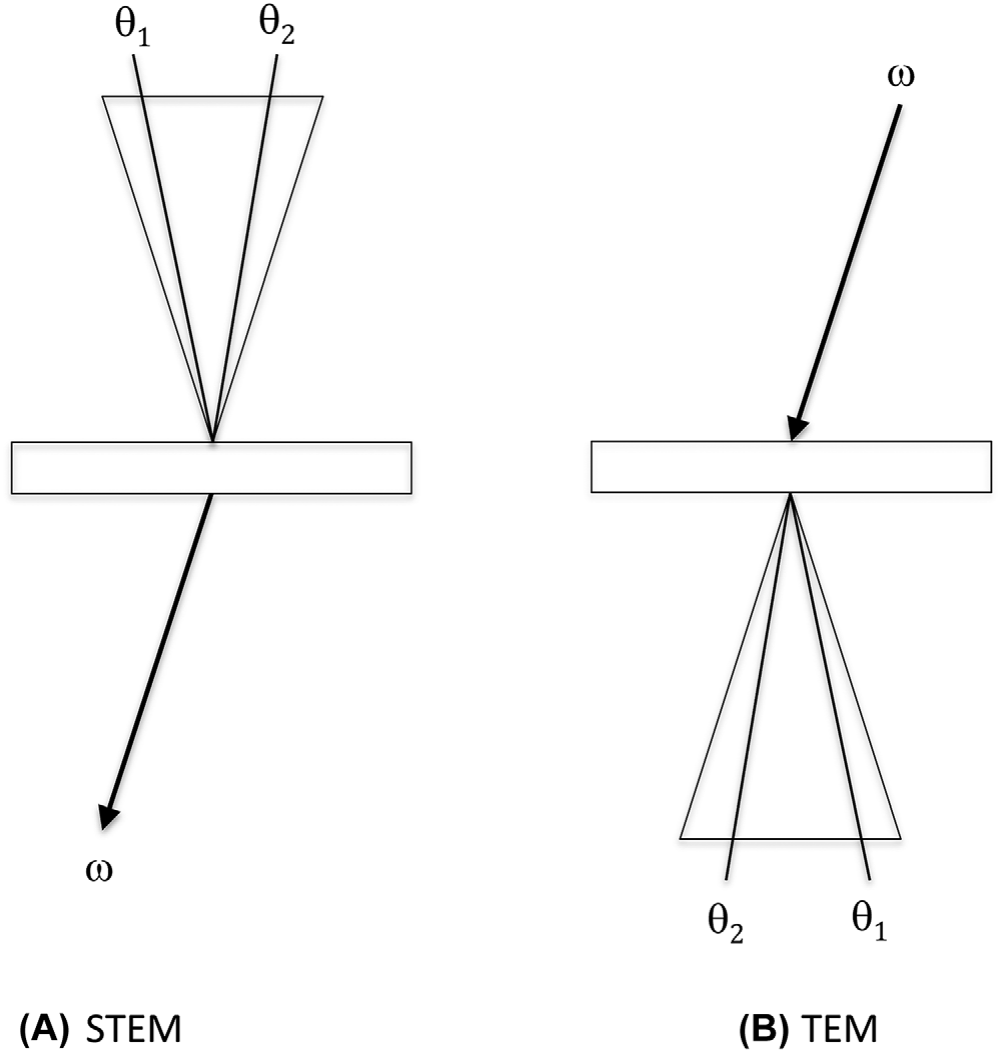
Analogy between imaging with detector element in STEM and tilted beam TEM.

Phase contrast in STEM with a field emission point source can be related to phase contrast in TEM by reciprocity as shown in Figure 5. The phase contrast arises from interference between scattering from different directions *θ*_1_ and *θ*_2_ in the illumination cone. The scattered electrons leave the specimen at an angle *ω* and form the signal received by a detector element. In its simplest form the detector could be a quadrant used for differential phase contrast, but in modern practice it is advantageous to use multi element direct electron detectors to collect the complete angular scattering distribution for each probe position, a practice known as 4D STEM. By reciprocity the scattering angle *ω* is equivalent to an illumination tilt, and the image observed by STEM is the same as the image for tilted illumination in TEM. Rose derived the contrast transfer function for a weak phase object for tilted illumination.^23^ To calculate the image for specimens that are not weak phase objects we will consider a specimen whose amplitude at the exit surface is the complex function *A*(**θ**), where *θ* is the scattering angle in the specimen. For simplicity, we will assume that *A*(**θ**) does not depend strongly on illumination angle *ω*, similar to the treatment of beam convergence in high resolution electron microscopy in materials science. The wave function after allowing for phase shifts from lens aberrations is

(9) $$\psi \;\left(r\right)=\sum_{\theta }A\left(\theta \right)\;\exp\left(i\chi \left(\theta +\omega \right)\right)\exp\left(i\frac{\theta }{\lambda }.r\right),$$

where χ(**θ**+ω) is the wavefront aberration function

(10) $$\chi \;\left(\theta +\omega \right)=\pi \lambda \left(\frac{1}{2}\frac{C_{s}}{\lambda^{2}}{\left|\theta +\omega \right|}^{4}-\frac{\Delta f}{\lambda^{2}}{\left|\theta +\omega \right|}^{2}\right).$$

The image intensity is

(11) $$\begin{array}{ccc} I\;\left(r\right) & = & \sum_{\theta_{1}\theta_{2}}A\left(\theta_{1}\right)\;A^{*}\left(\theta_{2}\right)\exp\left(i\chi \left(\theta_{1}+\omega \right)\right.\\ & & \left.-\;i\chi \left(\theta_{2}+\omega \right)\right)\exp\left(i\frac{\left(\theta_{1}-\theta_{2}\right)}{\lambda }.r\right). \end{array}$$

Expanding the phase shift term gives

(12) $$\begin{array}{c} \chi \left(\theta_{1}+\omega \right)-\chi \;\left(\theta_{2}+\omega \right)=\pi \lambda \;\times \\ \left[\left(\frac{1}{2}\frac{C_{s}}{\lambda^{2}}\theta_{1}^{4}-\frac{\Delta f}{\lambda^{2}}\theta_{1}^{2}\right)-\left(\frac{1}{2}\frac{C_{s}}{\lambda^{2}}\theta_{2}^{4}-\frac{\Delta f}{\lambda^{2}}\theta_{2}^{2}\right)\right.\\ \begin{array}{c} +\;2\left(\frac{C_{s}}{\lambda^{2}}\omega^{2}-\frac{\Delta f}{\lambda^{2}}\right)\left(\left(\theta_{1}-\theta_{2}\right).\omega \right)\\ +\frac{2C_{s}}{\lambda^{2}}\left(\left(\theta_{1}.\omega \right)\theta_{1}^{2}-\left(\theta_{2}.\omega \right)\theta_{2}^{2}\right)\\ \left.+\frac{4C_{s}}{\lambda^{2}}\left({\left(\theta_{1}.\omega \right)}^{2}-{\left(\theta_{2}.\omega \right)}^{2}\right)\right]. \end{array} \end{array}$$

The contribution $2\pi \lambda (\frac{C_{s}}{\lambda^{2}}\omega^{2}-\frac{\Delta f}{\lambda^{2}})\omega$ in the term with $(\theta_{1}-\theta_{2}).\omega$ is the phase shift giving rise to an image shift $(C_{s}\omega^{2}-\Delta f)\omega ,$ which would be corrected when summing the contributions from the different scattering angles corresponding to individual pixels. It can therefore be neglected in calculations of the phase contrast STEM signal. The 3rd and 4th terms multiplying Cs are higher order in angle and can be neglected when examining thick samples with low beam convergence. As a result, tilt corrected bright field effectively gives the same image as phase contrast TEM while using convergent illumination.^31^

### 1.3 Inelastic scattering

In TEM, the electrons that have been inelastically scattered do not focus to a unique plane, due to chromatic aberration in the objective lens. This effect contributes an out of focus background for any image, so in practice a narrow range of energies is selected with an energy filter. Normally this coincides with the zero‐loss peak, but imaging with other loss windows has been demonstrated for thick samples.^32^ Clearly it is desirable to maximise the signal, especially for cryo‐microscopy. In order to determine the optimal energy window and what proportion of the incident electrons may actually be used to form an image, we recorded energy loss spectra from different thicknesses of vitreous ice.

The spectra were recorded at 200 kV acceleration on a TFS Themis‐Z microscope in STEM microprobe mode, with a beam convergence semi angle of 0.47 mrad and a probe current of about 15 pA. The semi‐collection angle was 17 mrad, and the spectra were were extracted from 1028 × 130 pixels on the DECTRIS ELA detector with 0.5 eV/channel covering a range of 512 eV. The thickness in terms inelastic mean free path, *λ_imfp_*, was estimated according to equation

(13) $$\;\frac{t}{\lambda_{imfp}}=\;\ln\left(\frac{I_{T}}{I_{0}}\right),$$

where *I_T_* is the total intensity in the spectrum and *I*_0_ is the zero loss intensity.^11^ Additionally, the probe current was increased using a 150 µ condenser aperture corresponding to 1.4 mrad convergence semi angle, and holes drilled through the vitreous ice. The thickness was estimated independently from the length of the hole measured in the image when the specimen was tilted by 25°.

**FIGURE 6:**
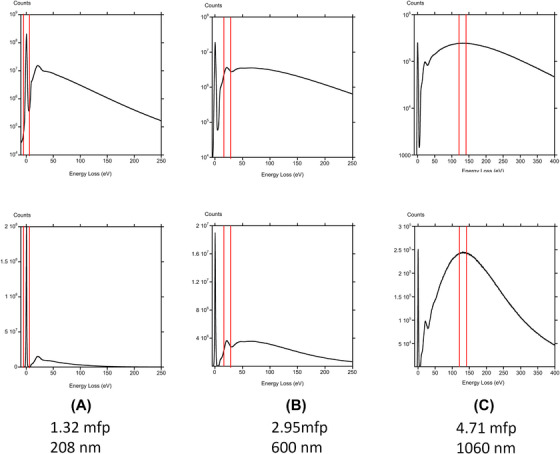
EELS spectra for 200 keV electrons from various thicknesses of vitreous ice: (A) 1.32 mfp 208 nm, (B) 2.95 mfp 600 nm, (C) 4.71 mfp 1070 nm. In the upper row the spectra are plotted on a log scale so that all the features are visible, in the lower row the prominent features are shown on a linear scale.

Below 230 nm, as shown in Figure 6A, most of the intensity is in the zero loss peak. For greater thicknesses up to about 600 nm (Figure 6B) the plasmon peak at about 20 eV dominates. For very thick specimens whose thickness is almost 1 micron, the spectrum takes the form of a Landau distribution, Figure 6C, whose peak at about 130 eV is the most prominent feature. The thickness estimates in terms of inelastic mean free path might not be accurate for the thicker regions as there will be inelastic scattering outside the EELS collection aperture. We have therefore estimated the inelastic mean free path as 157 nm from the thinnest region shown as Figure 6A, which is 0.27 the elastic mean free path.^26^

**FIGURE 7:**
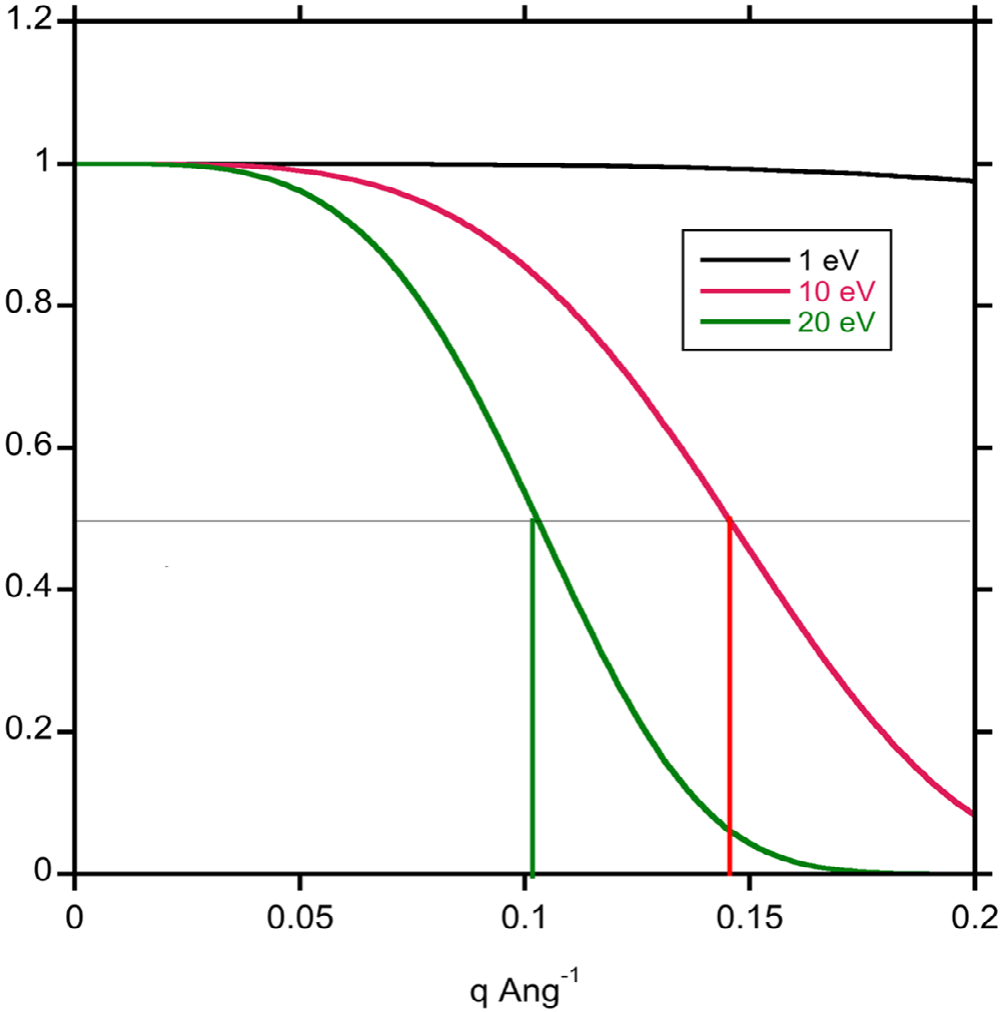
Temporal coherence envelope for Gaussians of different half width.

The range of energies in windows that select plasmon or higher energy losses will also decrease the temporal coherence. For the zero loss peak, the narrow energy spread of the FEG source determines the temporal coherence and resulting decay in the contrast transfer function for imaging with the zero loss peak. This is not the case for imaging using the plasmon, whose half width is about 10 eV, or the peak in the Landau distribution if a 20 eV window is chosen. Figure 7 shows the temporal coherence function^33^ that acts as an aperture in phase contrast imaging. Spatial resolution due to temporal coherence is limited to 0.6 nm for imaging using the plasmon peak, and 1.0 nm imaging using the peak of the Landau distribution.

**FIGURE 8:**
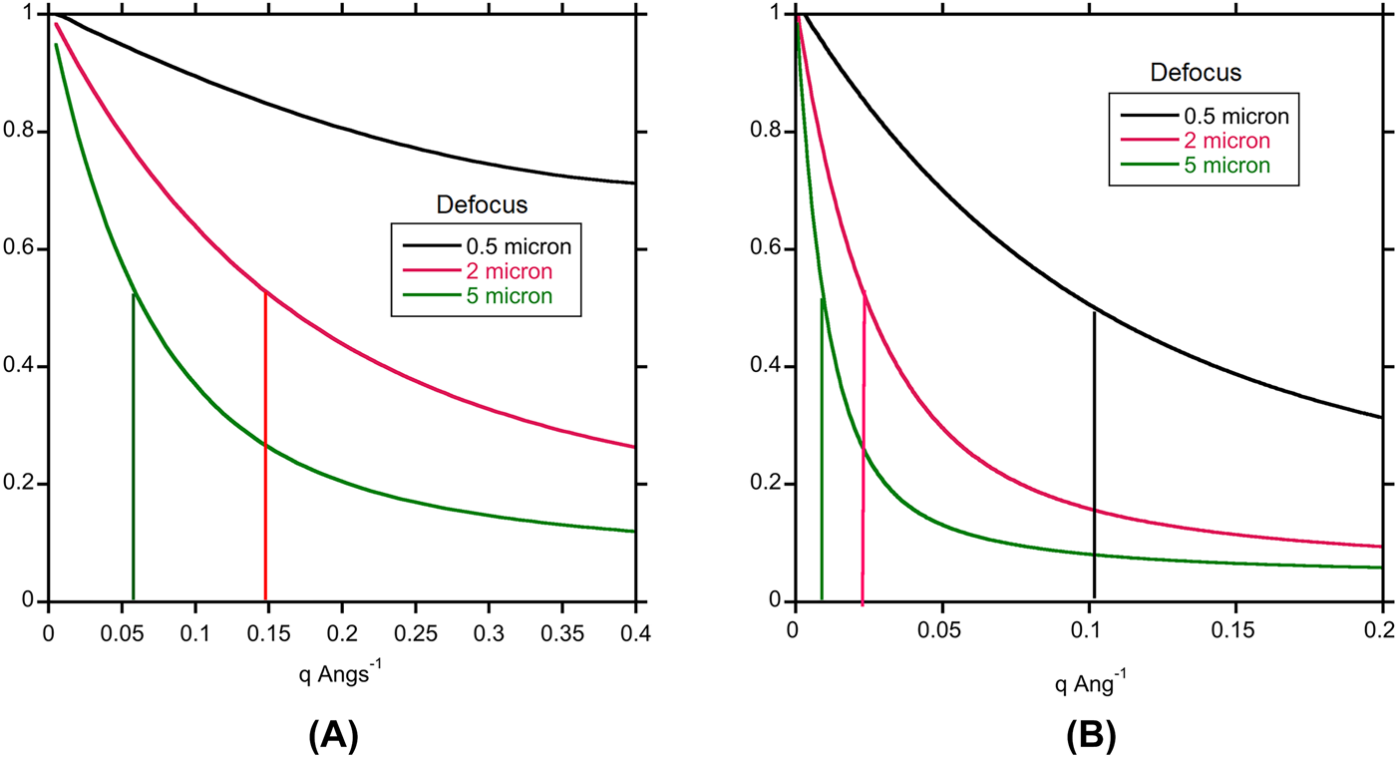
Spatial coherence envelopes for Lorentzians appropriate for (A) 20 eV loss and (B) 130 eV loss for defocus values of 0.5 µm, 2 µm and 5µm.

The degradation in spatial coherence from the angular spread of the inelastically scattered electrons should also be considered. The intensity of inelastic scattering is greater than the elastic scattering up to about 7 mrad at 200 keV,^13^ and, as pointed out, there is more scattering to high angles with a Lorentzian distribution that emerges from dipole scattering by the plasmon than in the Gaussian distribution that was used to represent a broad source in the original derivation of spatial coherence.^28^ The spatial coherence function is shown in Figure 8 for a plasmon loss of 20 eV and a loss corresponding to the peak of the Landau distribution at 130 eV. Given the dependence on $\frac{d\chi }{d\vartheta }$ it is not surprising that the spatial resolution at 2 µm and 5 µm defocus is subject to severe limitations. At 5 µm defocus the spatial resolution, given by when the spatial coherence function drops by a factor of 2, is about 2 nm for imaging in the plasmon peak and 10 nm in the peak of the Landau distribution. In practice these might not be the primary limitations, given the thickness of the specimens where these energies would be selected. More significant is the loss of signal. For the cases shown in Figure 6B anD C, only about 8%–9% of the incident electrons are being used to form an image for a 20 eV energy selecting windows. The more than 90% of incident electrons that don't give useful imaging information are still damaging the specimen. For vitreous ice on its own it becomes optimal to use the plasmon for imaging for thicknesses greater than 230 nm. From this analysis it would seem that attempting to image by EFTEM using the peak of the Landau distribution is not worthwhile, and is even marginal for imaging in the plasmon peak.

In principle a chromatic aberration corrector that recovers phase contrast in the presence of inelastic scattering should be equivalent to STEM,^34^ but an analysis of spatial resolution should also consider the additional contributions from spatial and temporal coherence. The authors point out that the deleterious effects from a reduction in spatial coherence may be avoided by operating with a phase plate at zero defocus. This could greatly benefit studies of molecular structure, but again limits the specimen thickness.

### 1.4 Simulated images for a model phage

**FIGURE 9:**
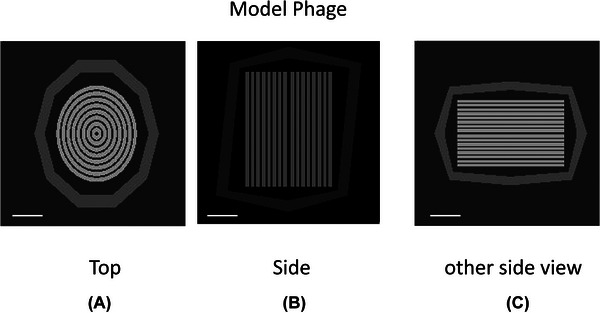
Cross‐sections of the model T4 phage with view direction perpendicular to (A) top, (B) side, (C) other side. Bar is 20 nm.

To investigate how cryo‐ET and cryo‐STEM tomography compare at a theoretical level, we constructed a model of a T4 phage capsid using the Penelope geometry engine.^24^ The icosahedral capsid was defined by planes, and the DNA was arranged in concentric cylinders with a 1 nm gap between them. This arrangement of DNA was chosen as it provided a suitable geometry for testing resolution, though we recognise it is probably not an accurate description of the structure.^35^ The relative volume of the capsid and DNA was taken from the data given by Yap and Rossman.^36^ A listing of the Penelope geometry engine commands is given in the Supplementary Information. The 3 orthogonal views of the model phage are shown as Figure 9.

To calculate the CSTET images from incoherent scattering we used the Penelope Monte Carlo code, following the same procedure as was used in Rez et al.^26^ Monte Carlo codes use random numbers to sample scattering angles and energy losses from integrated cross sections. The scattered electrons are followed along their new direction of travel for a random distance till they experience another scattering event. The angular distribution of scattered electrons is built up by following multiple trajectories. Due to limitations in the sampling of both angular distributions and path lengths the Penelope code is not suitable for vitreous ice specimens thinner than about 300 nm. In practice this might not be a serious limitation as incoherent scattering does not make a large contribution to image contrast for thinner specimens. In our calculations 10,000 electrons were incident on 1 nm pixels and the number that reached detectors in the range 0–7 mrad (bright field), 10–20 mrad, 20–30 mrad and 30–44 mrad were tabulated. These corresponded to the detector segments of the OPAL segmented STEM detector (El Mul Technologies Ltd.). For 1 MeV the scattering angles change by the ratio of electron wavelengths and the detector segments were changed to 0–2.43 mrad, 3.5–7.0 mrad, 7.0–10.4 mrad and 10.4–15.3 mrad. The electrons per pixel matched the electron exposure that might be used in CTSET (100 e^−^/Å^2^) and the probe was stepped over a 110×110 grid giving a field of view of 110×110 nm corresponding roughly to the capsid size.

The calculations for TEM contrast used a slightly larger cell with dimensions 128 nm × 128 nm. This was sampled with 512×512 points for convenience of performing the Fast Fourier Transform needed for the multislice calculation.^37^ The phase gratings were calculated from the average electron scattering factors given in Table 3 using Equations (3) and (4), where it was assumed that electrons scattered beyond 6 mrad were ‘absorbed’. This corresponds to a spatial resolution of 0.42 nm. The electron accelerating voltage was chosen as 200 keV, with a *C_s_* of 2 mm, typical of an FEI F20 and the objective aperture semi‐angle was 4 mrad. To simulate the loss of electrons from the zero loss due to inelastic scattering the fluence was reduced to 28% of the incident fluence, since the 200 nm ice thickness corresponded to 1.27 inelastic mean free paths. It was assumed that the detector was almost perfect and that the only noise came from Poisson statistics of electrons arriving in a given pixel, simulated by random sampling from a Poisson distribution.

**TABLE 3:** Real and imaginary parts of the electron scattering factor used for multislice TEM contrast calculations.

	Average *f*_el_ real	Average *f*_el_ image	Elastic mfp
Vitreous ice	0.8885	1.82 × 10^−4^	581 nm
Protein	1.5170	2.71 × 10^−4^	342 nm
DNA/RNA	1.5808	3.62 × 10^−4^	256 nm

A beam convergence of 3 mrad was chosen for the tilt corrected bright‐field STEM images, since this gave a depth of focus of 277 nm, greater than the 200 nm thickness of vitreous chosen for embedding the phage. It was assumed the detector covered an angular range of 4 mrad and was divided into 20 annular rings with 90 azimuthal segments (4° slices). A modification of the TEM multislice code was used with the wavefront aberration function given by Equation (12) without the tilt contribution. Since there is no energy selection there was no reduction in electron fluence.

## 2 RESULTS AND DISCUSSION

**FIGURE 10:**
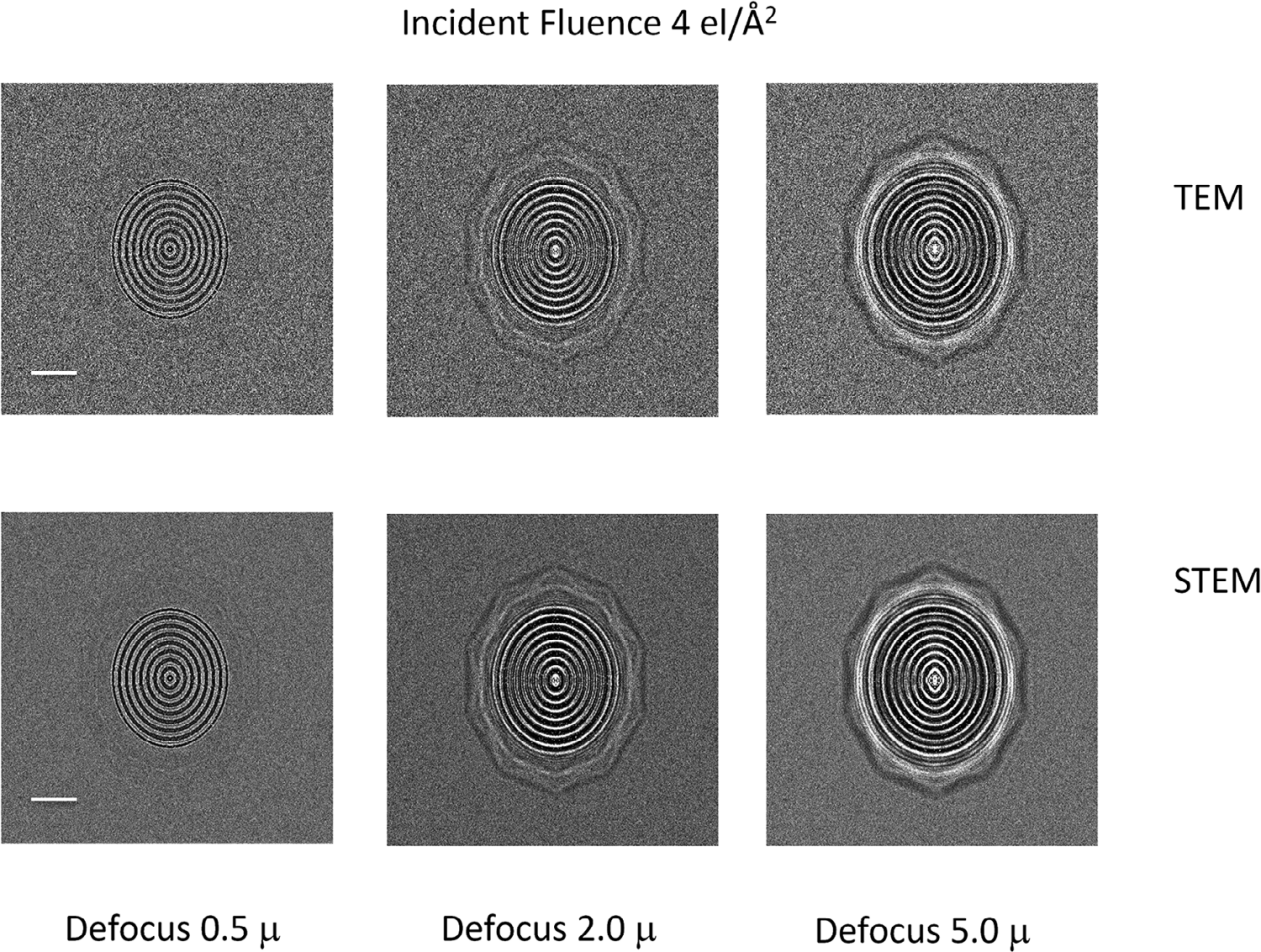
Multislice simulated phase contrast 200 keV TEM (top row) and STEM (bottom row) images for the phage in the orientation shown in Figure 9A in the middle of 200 nm of vitreous ice for defocus values of 0.5 µ, 2.0 µ and 5.0 µ. The incident fluence was 4 electrons/Å^2^. The reduced S/N for the TEM images is a consequence of the reduction in number of electrons from selecting the zero loss. Bar is 20 nm. The maximum intensity is 15 electrons per 2.5 Å square pixel, with a noise level of about 5 electrons for the TEM images and 2 electrons for the STEM images.

**FIGURE 11:**
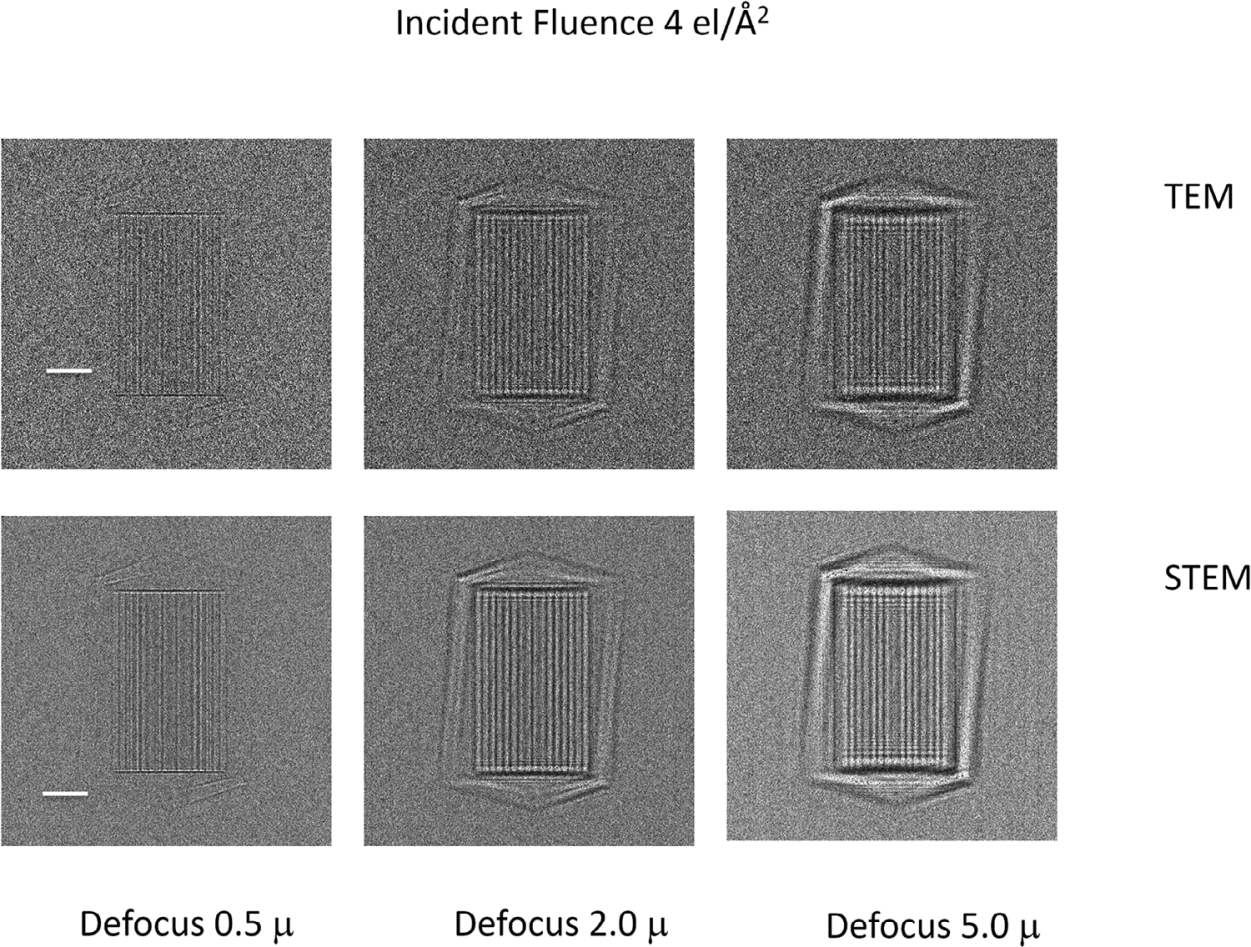
Multislice simulated 200 keV TEM (top row) and STEM (bottom row) for the phage in the orientation shown in Figure 9B in the middle of 200 nm of vitreous ice for defocus values of 0.5 µm, 2.0 µm and 5.0 µm. The incident fluence was 4 electrons/Å^2^. The reduced S/N for the TEM images is a consequence of the reduction in the number of electrons from selecting the zero loss. Bar is 20 nm. The maximum intensity is 15 electrons per 2.5 Å square pixel, with a noise level of about 5 electrons for the TEM images and 2 electrons for the STEM images.

The simulated phase contrast images for both TEM and STEM at a defocus value of 0.5 µm, as well as defocus values of 2 µm and 5 µm, frequently used for picking out the overall shapes of objects at low resolution, are shown in Figure 10 for the orientation of Figure 9A and Figure 11 for the orientation shown in Figure 9C. Correcting the image shift for the detector pixels in STEM means that the contrast will be identical to that observed in the TEM, and since the range of angles collected by the detector is matched to the beam convergence, the collection efficiency is optimised. The angle subtended by an individual detector pixel is equivalent to the beam convergence in TEM whose effect can be represented by the spatial coherence envelope function. The calculations show the images for an electron fluence of 4 electrons/Å^2^. Since the zero loss only has 28% of the incident electrons as estimated from the inelastic mean free path, the TEM images are noisier than the STEM images.

The contrast in STEM is much improved from conventional TEM imaging, in agreement with the results of Yu et al.^22^ This is a consequence of making use of all electrons, including those that have been inelastically scattered. Given that bright field is more favourable for high resolution imaging even for incoherent scattering^26^, ^38^, ^39^, ^40^ the theory of Du and Jacobsen^41^ can be used to give a first order estimate of electron fluence and contrast.

**FIGURE 12:**
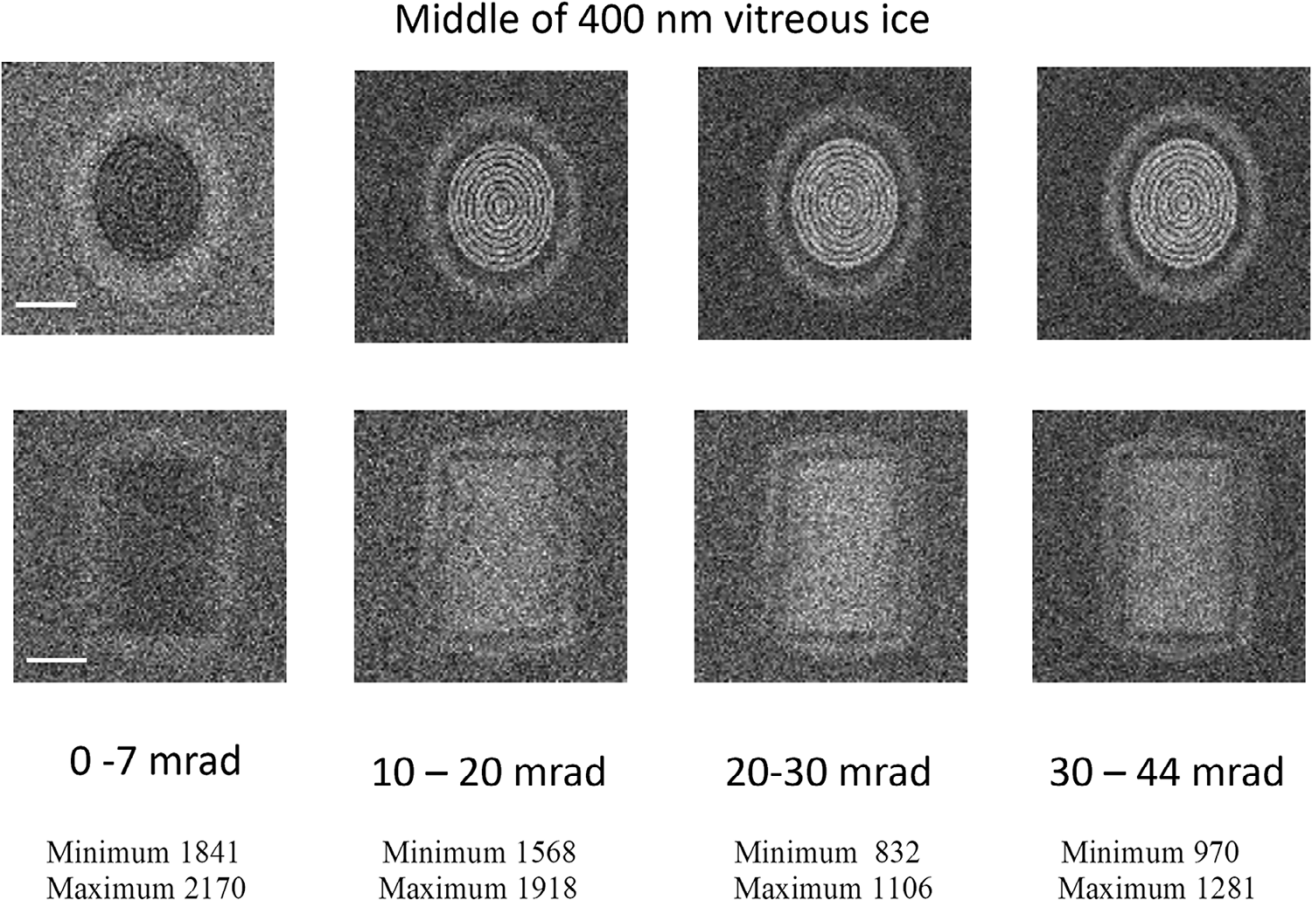
Monte Carlo simulated 200 keV incoherent STEM images for the phage in the orientation shown in Figure 9A (top row) and the orientation shown in Figure 9C (bottom row) in the middle of 400 nm vitreous ice for collection angles 0–7 mrad (bright field), 10–20 mrad, 20–30 mrad and 30–44 mrad (dark field). Bar is 25 nm. The minimum and maximum number of electrons in a 1 nm square pixel is given below the images.

Figure 12 shows the incoherent STEM contrast for the phage as oriented in Figure 9A and C in the middle 400 nm of vitreous ice. In all case except the bright‐field image the concentric cylinders of the DNA can be clearly seen. As expected there is a change in contrast for bright field (0–7 mrad) as opposed to dark field (other detector angles). For the orientation shown as Figure 9C, the side view, there is a projection through the phage, and the thickness of both DNA and capsid protein along the electron path length is much reduced. As a consequence there is lower contrast as can be seen in second row of Figure 12. Calculations were also performed for both orientations with the phage near the entrance surface at a depth of 100 nm as well as near the exit surface at a depth of 300 nm in 400 nm of vitreous ice. No discernible differences in contrast as shown in Figures S2 and S3 were seen, confirming the absence of a top‐bottom effect in the dark‐field.

**FIGURE 13:**
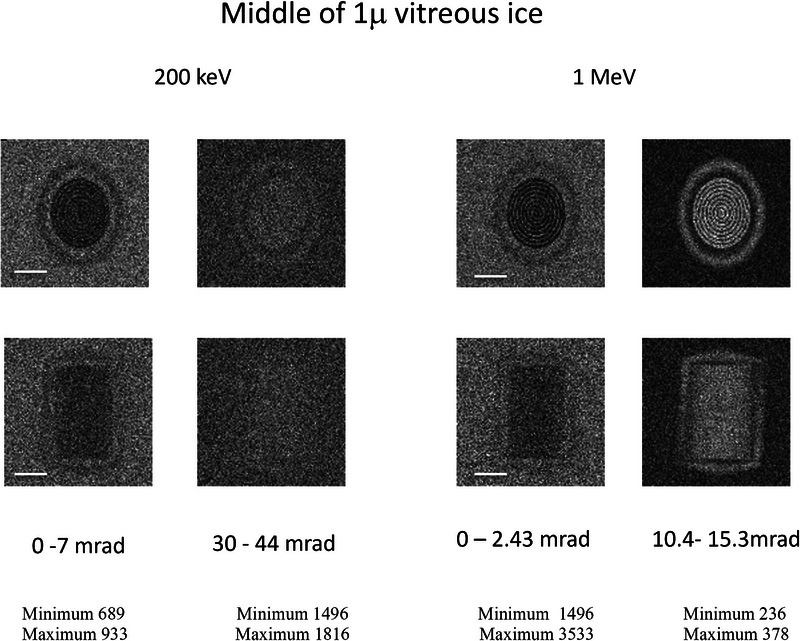
Comparison of Monte Carlo simulated 200 keV and 1 MeV incoherent STEM images for the phage in the orientation specified by Figure 9A (top row) and Figure 9B (bottom row). The angular ranges of the bright‐field and dark‐field detectors were chosen to cover the same range of wavevectors. The bright‐field detector spans a range of 0–7 mrad at 200 keV and 0–2.43 mrad at 1 MeV. The dark‐field detector spans the range from 30–44 mrad at 200 keV and 10.4 to 15.4 mrad at 1 MeV. The minimum and maximum number of electrons in a 1 nm square pixel is given below the images.

Ultimately our goal is to image features in an organelle or prokaryotic cell about 1 micron thick. This is about double the elastic mean free path in vitreous ice, so the angular distribution will be broadened by multiple scattering. Although the phage is small compared to structures that might be present in a prokaryotic cell we might still be able to gain some insight as to what is possible for imaging in thicker specimens when we consider the phage at the centre of 1 m of vitreous ice. Figure 13 shows the simulation for a bright‐field and dark‐field detectors for 200 keV and 1 MeV. As shown in earlier work,^26^, ^38^, ^39^, ^40^ bright field is superior for very thick specimens as the contribution from large angle scattering events that degrade spatial resolution is much reduced. It would appear that 1 MeV would be a better choice for the accelerating voltage since the key features are still discernible in the dark‐field detector. This is not so much a consequence of increased path length or penetration as much as a reduction in scattering angles that minimises the number of resolution‐degrading large angle events.

## 3 CONCLUSIONS

The contrast in biological specimens arises from the difference in scattering between protein, genetic material and vitreous ice. Image interpretation in phase contrast TEM is based on a weak phase object approximation, from which a contrast transfer function can be derived. The fidelity of high‐resolution information depends on correction of the contrast inversions so described. Objects whose thickness exceeds about 50 nm are not weak phase objects even if the accelerating voltage is increased from 300 keV to 1 MeV and beyond. In practice, thicker biological specimens, up to about 200 nm in thickness, behave as a combination of amplitude and phase objects where the amplitude contrast comes from scattering to higher angles, outside the angular range used for phase contrast. In TEM, the limited spatial and temporal coherence impose an effective aperture, even without a physical aperture in the optical path. Moreover, it is necessary to select the zero loss or a narrow range of energies to avoid the deleterious effects of chromatic aberration. For thick biological specimens, selecting either plasmon scattered electrons or electrons from the peak in the Landau distribution means that 90% of the incident electrons are not giving any information but still contribute to radiation damage.

It is also possible for phase contrast to contribute to images formed in STEM. Tilt corrected bright‐field images using pixelated detectors matching the incident beam convergence make efficient use of all the incident electrons. Since there is no energy filtering the only loss of electrons is to large angle elastic scattering. The elastic mean free path, which is about 3 times greater than the inelastic mean free path, sets a limit of about 600 nm for specimen thickness. STEM has a significant advantage over TEM for phase contrast in thicker specimens as selecting a range of energies in TEM inevitably means throwing away signal.

For the thickest specimens, incoherent amplitude contrast dominates. It is in principle possible to optimise the signal to noise or contrast by varying the range of scattering angles that are collected using STEM with pixelated detectors. This can even be done in a limited way with fixed annular detectors by changing the camera length.

Using higher accelerating voltages in the MeV range does not make much difference to the path length or ‘penetration’, which saturates around 700 keV. However there might still be some improvements in bright‐field resolution from the reduction in scattering angles.

## Supporting information



SUPPORTING INFORMATION
