# Supplementary material for: Contrast by electron microscopy in thick biological specimens
Source: J Microsc. 2025 Aug 26;300(3):341–55. doi: 10.1111/jmi.70026 (PMC12637017; doi:10.1111/jmi.70026)
Supplement: Supplementary file 1 — SUPPORTING INFORMATION [file JMI-300-341-s001.pdf]

Supplementary Information for:

Contrast by Electron Microscopy in Thick Biological Specimens

Peter Rez, Lothar Houben, Shahar Seifer, Michael Elbaum

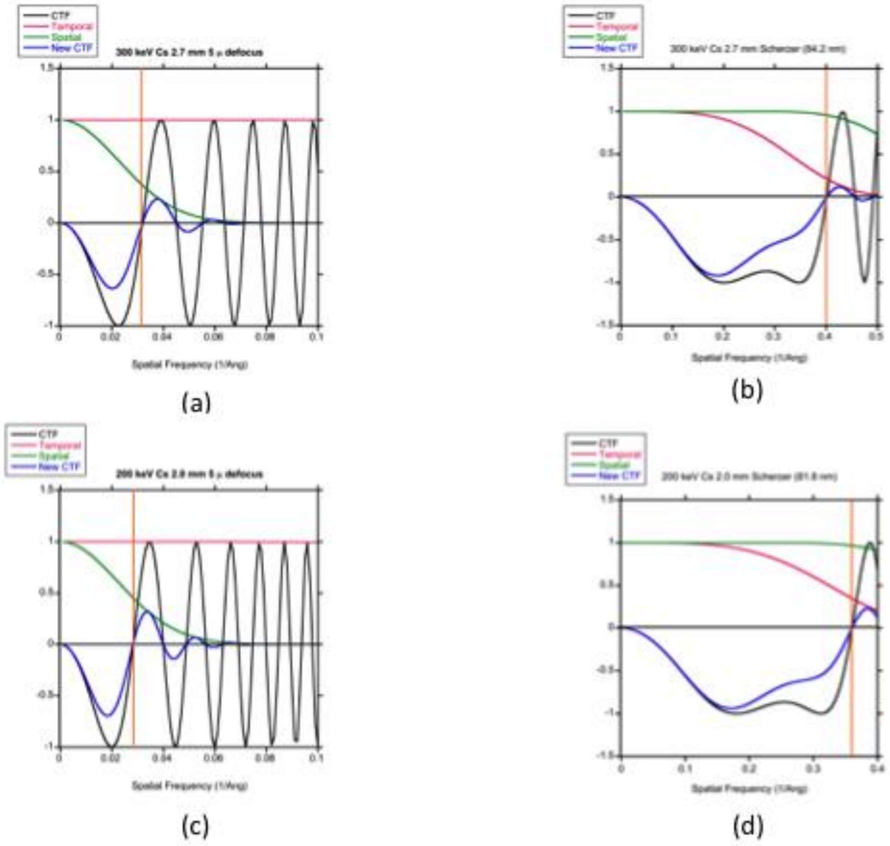

Supplementary Figure 1

Comparison of contrast transfer functions for Krios 300 keV, Cs=2.7 mm and a FEI F20 200 keV Cs=2.0 mm showing spatial frequency range with limit (orange line) for (a) 5 μm defocus for (a) Krios and (c) F 20 and Scherzer focus for (b) Krios (defocus 84.2 nm, spread 25nm) and (d) FEI F20 (defocus 81.8 nm, spread 20nm) all with focal spread 50 nm and beam divergence 0.1 mrad.

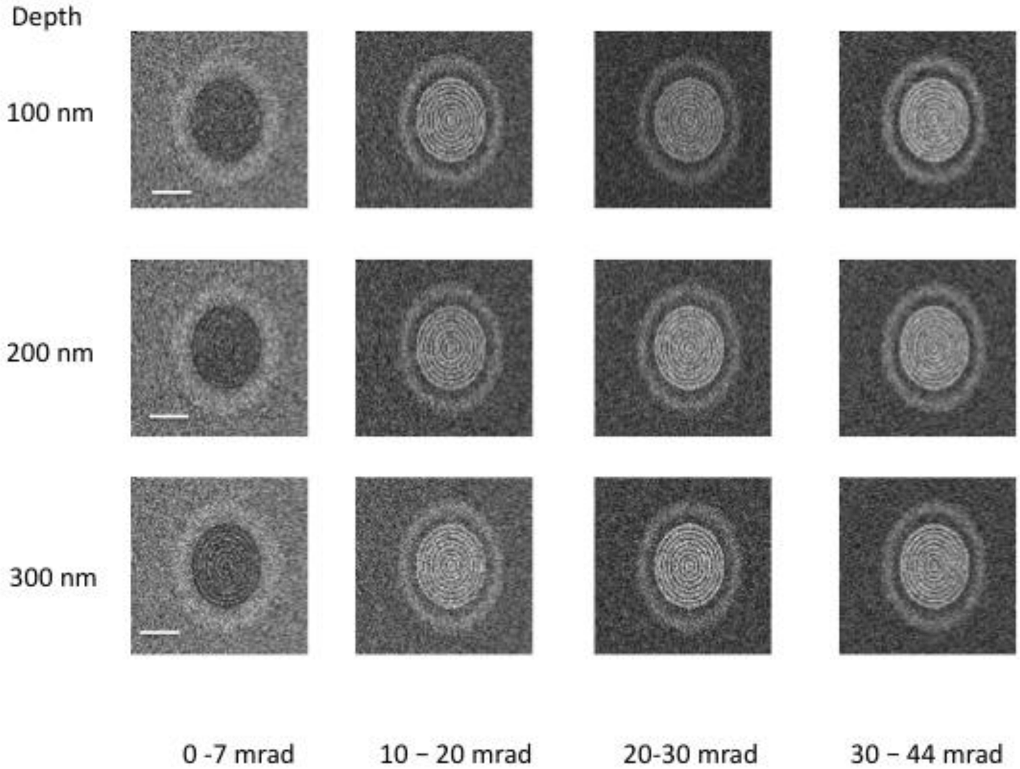

Supplementary Figure 2

Monte Carlo simulated 200 keV incoherent STEM images for the phage in the orientation shown in Fig 9a at a depth of 100 nm (top row), 200 nm (middle row) and 300 nm (bottom row) in 400 nm vitreous ice for collection angles 0-7 mrad (Bright Field), 10-20 mrad , 20-30 mrad and 30-44 mrad (dark field) Bar is 25 nm

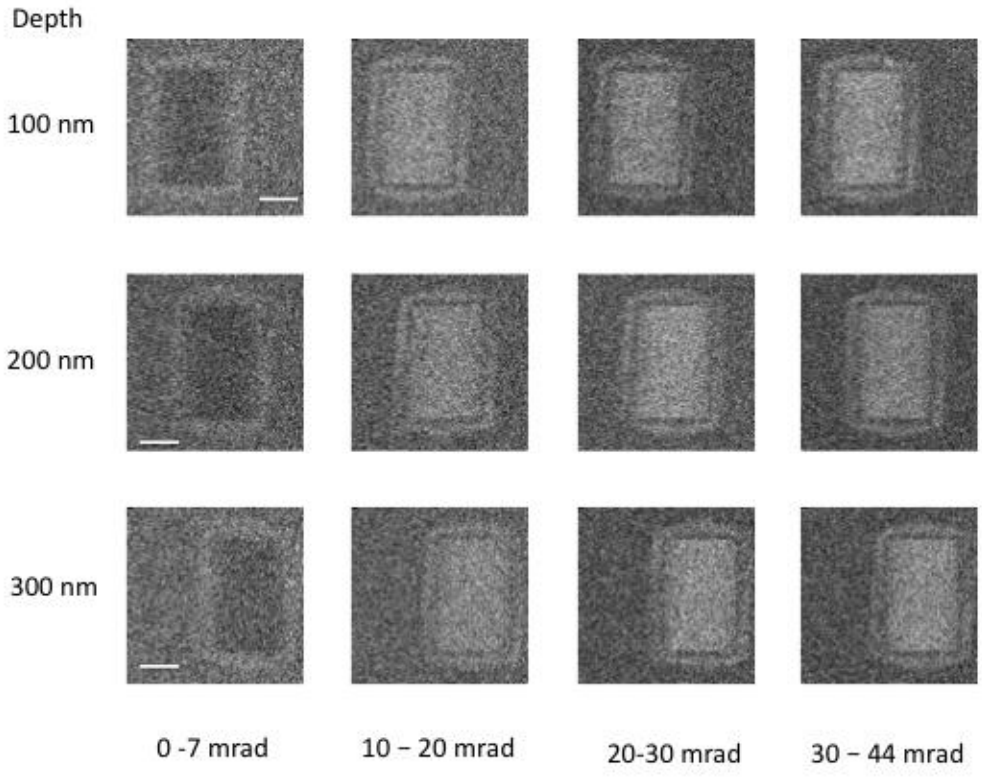

Supplementary Figure 3

Monte Carlo simulated 200 keV incoherent STEM images for the phage in the orientation shown in Fig 9c at a depth of 100 nm (top row), 200 nm (middle row), and 300 nm (bottom row) in 400 nm vitreous ice for collection angles 0-7 mrad (Bright Field), 10-20 mrad, 20-30 mrad, and 30-44 mrad (dark field) Bar is 25 nm

Penelope Geometry Engine Commands for Phage in Orientation Shown in Fig 9a

```
0000000000000000000000000000000000000000000000000
SURFACE (  1)
INDICES=( 0, 0, 0, 0, 0)
    AXX=( 0.000000000000000E+00,  0)
    AXY=( 0.000000000000000E+00,  0)
    AXZ=( 0.000000000000000E+00,  0)
    AYY=( 0.000000000000000E+00,  0)
    AYZ=( 0.000000000000000E+00,  0)
    AZZ=( 0.000000000000000E+00,  0)
     AX=( 0.592833369868559E-01,  0)
     AY=( 0.225534221310335E+00,  0)
     AZ=( 0.972429843728401E+00,  0)
     A0=( 4.222011833478610E-06,  0)
0000000000000000000000000000000000000000000000000
SURFACE (  2)
INDICES=( 0, 0, 0, 0, 0)
    AXX=( 0.000000000000000E+00,  0)
    AXY=( 0.000000000000000E+00,  0)
    AXZ=( 0.000000000000000E+00,  0)
    AYY=( 0.000000000000000E+00,  0)
    AYZ=( 0.000000000000000E+00,  0)
    AZZ=( 0.000000000000000E+00,  0)
     AX=(-0.156060836802792E+00,  0)
     AY=( 0.140154446780401E+00,  0)
     AZ=( 0.977753417925037E+00,  0)
     A0=( 4.209392845810320E-06,  0)
0000000000000000000000000000000000000000000000000
SURFACE (  3)
INDICES=( 0, 0, 0, 0, 0)
    AXX=( 0.000000000000000E+00,  0)
    AXY=( 0.000000000000000E+00,  0)
    AXZ=( 0.000000000000000E+00,  0)
    AYY=( 0.000000000000000E+00,  0)
    AYZ=( 0.000000000000000E+00,  0)
    AZZ=( 0.000000000000000E+00,  0)
     AX=(-0.156060944481120E+00,  0)
     AY=(-0.140149620425506E+00,  0)
     AZ=( 0.977754092552032E+00,  0)
     A0=( 4.209391822847660E-06,  0)
0000000000000000000000000000000000000000000000000
SURFACE (  4)
INDICES=( 0, 0, 0, 0, 0)
    AXX=( 0.000000000000000E+00,  0)
    AXY=( 0.000000000000000E+00,  0)
    AXZ=( 0.000000000000000E+00,  0)
    AYY=( 0.000000000000000E+00,  0)
    AYZ=( 0.000000000000000E+00,  0)
    AZZ=( 0.000000000000000E+00,  0)
     AX=( 0.592838567020995E-01,  0)
     AY=(-0.225536198486027E+00,  0)
     AZ=( 0.972429353478697E+00,  0)
     A0=( 4.222012481265670E-06,  0)
0000000000000000000000000000000000000000000000000
SURFACE (  5)
```

```
INDICES=( 0, 0, 0, 0, 0)
    AXX=( 0.000000000000000E+00,  0)
    AXY=( 0.000000000000000E+00,  0)
    AXZ=( 0.000000000000000E+00,  0)
    AYY=( 0.000000000000000E+00,  0)
    AYZ=( 0.000000000000000E+00,  0)
    AZZ=( 0.000000000000000E+00,  0)
     AX=( 0.193559781656466E+00,  0)
     AY=(-0.493976576298720E-05,  0)
     AZ=( 0.981088482707192E+00,  0)
     A0=( 4.200915240447980E-06,  0)
0000000000000000000000000000000000000000000000000000000000000000
SURFACE (   6)
INDICES=( 0, 0, 0, 0, 0)
    AXX=( 0.000000000000000E+00,  0)
    AXY=( 0.000000000000000E+00,  0)
    AXZ=( 0.000000000000000E+00,  0)
    AYY=( 0.000000000000000E+00,  0)
    AYZ=( 0.000000000000000E+00,  0)
    AZZ=( 0.000000000000000E+00,  0)
     AX=( 0.253393311097366E+00,  0)
     AY=( 0.963995382315658E+00,  0)
     AZ=( 0.806519235059030E-01,  0)
     A0=( 2.724565092389150E-06,  0)
0000000000000000000000000000000000000000000000000000000000000000
SURFACE (   7)
INDICES=( 0, 0, 0, 0, 0)
    AXX=( 0.000000000000000E+00,  0)
    AXY=( 0.000000000000000E+00,  0)
    AXZ=( 0.000000000000000E+00,  0)
    AYY=( 0.000000000000000E+00,  0)
    AYZ=( 0.000000000000000E+00,  0)
    AZZ=( 0.000000000000000E+00,  0)
     AX=(-0.995197837681966E+00,  0)
     AY=( 0.253980664986645E-04,  0)
     AZ=(-0.978839273225069E-01,  0)
     A0=( 3.307920021502780E-06,  0)
0000000000000000000000000000000000000000000000000000000000000000
SURFACE (   8)
INDICES=( 0, 0, 0, 0, 0)
    AXX=( 0.000000000000000E+00,  0)
    AXY=( 0.000000000000000E+00,  0)
    AXZ=( 0.000000000000000E+00,  0)
    AYY=( 0.000000000000000E+00,  0)
    AYZ=( 0.000000000000000E+00,  0)
    AZZ=( 0.000000000000000E+00,  0)
     AX=(-0.740980502895301E+00,  0)
     AY=( 0.665456591070231E+00,  0)
     AZ=( 0.900856244370604E-01,  0)
     A0=( 3.043783683849670E-06,  0)
0000000000000000000000000000000000000000000000000000000000000000
SURFACE (   9)
INDICES=( 0, 0, 0, 0, 0)
    AXX=( 0.000000000000000E+00,  0)
    AXY=( 0.000000000000000E+00,  0)
    AXZ=( 0.000000000000000E+00,  0)
```

```
    AYY=( 0.000000000000000E+00,  0)
    AYZ=( 0.000000000000000E+00,  0)
    AZZ=( 0.000000000000000E+00,  0)
     AX=(-0.253393311097366E+00,  0)
     AY=(-0.963995382315658E+00,  0)
     AZ=(-0.806519235059030E-01,  0)
     A0=( 2.724565092389150E-06,  0)
0000000000000000000000000000000000000000000000000000
SURFACE ( 10)
INDICES=( 0, 0, 0, 0, 0)
    AXX=( 0.000000000000000E+00,  0)
    AXY=( 0.000000000000000E+00,  0)
    AXZ=( 0.000000000000000E+00,  0)
    AYY=( 0.000000000000000E+00,  0)
    AYZ=( 0.000000000000000E+00,  0)
    AZZ=( 0.000000000000000E+00,  0)
     AX=(-0.740991792672244E+00,  0)
     AY=(-0.665443355009257E+00,  0)
     AZ=( 0.900905348324518E-01,  0)
     A0=( 3.043850240451650E-06,  0)
0000000000000000000000000000000000000000000000000000
SURFACE ( 11)
INDICES=( 0, 0, 0, 0, 0)
    AXX=( 0.000000000000000E+00,  0)
    AXY=( 0.000000000000000E+00,  0)
    AXZ=( 0.000000000000000E+00,  0)
    AYY=( 0.000000000000000E+00,  0)
    AYZ=( 0.000000000000000E+00,  0)
    AZZ=( 0.000000000000000E+00,  0)
     AX=( 0.740980502895301E+00,  0)
     AY=(-0.665456591070231E+00,  0)
     AZ=(-0.900856244370604E-01,  0)
     A0=( 3.043783683849670E-06,  0)
0000000000000000000000000000000000000000000000000000
SURFACE ( 12)
INDICES=( 0, 0, 0, 0, 0)
    AXX=( 0.000000000000000E+00,  0)
    AXY=( 0.000000000000000E+00,  0)
    AXZ=( 0.000000000000000E+00,  0)
    AYY=( 0.000000000000000E+00,  0)
    AYZ=( 0.000000000000000E+00,  0)
    AZZ=( 0.000000000000000E+00,  0)
     AX=( 0.253393311097366E+00,  0)
     AY=(-0.963995382315658E+00,  0)
     AZ=( 0.806519235059030E-01,  0)
     A0=( 2.724539735436340E-06,  0)
0000000000000000000000000000000000000000000000000000
SURFACE ( 13)
INDICES=( 0, 0, 0, 0, 0)
    AXX=( 0.000000000000000E+00,  0)
    AXY=( 0.000000000000000E+00,  0)
    AXZ=( 0.000000000000000E+00,  0)
    AYY=( 0.000000000000000E+00,  0)
    AYZ=( 0.000000000000000E+00,  0)
    AZZ=( 0.000000000000000E+00,  0)
     AX=( 0.740991792672244E+00,  0)
```

AY=(0.665443355009257E+00, 0)
AZ=(-0.900905348324518E-01, 0)
A0=(3.043850240451630E-06, 0)
0000000000000000000000000000000000000000000000
SURFACE (14)
INDICES=(0, 0, 0, 0, 0)
AXX=(0.000000000000000E+00, 0)
AXY=(0.000000000000000E+00, 0)
AXZ=(0.000000000000000E+00, 0)
AYY=(0.000000000000000E+00, 0)
AYZ=(0.000000000000000E+00, 0)
AZZ=(0.000000000000000E+00, 0)
AX=(0.995197837681966E+00, 0)
AY=(-0.253980664986645E-04, 0)
AZ=(0.978839273225069E-01, 0)
A0=(3.307920021502780E-06, 0)
0000000000000000000000000000000000000000000000
SURFACE (15)
INDICES=(0, 0, 0, 0, 0)
AXX=(0.000000000000000E+00, 0)
AXY=(0.000000000000000E+00, 0)
AXZ=(0.000000000000000E+00, 0)
AYY=(0.000000000000000E+00, 0)
AYZ=(0.000000000000000E+00, 0)
AZZ=(0.000000000000000E+00, 0)
AX=(-0.253393311097366E+00, 0)
AY=(0.963995382315658E+00, 0)
AZ=(-0.806519235059030E-01, 0)
A0=(2.724539735436340E-06, 0)
0000000000000000000000000000000000000000000000
SURFACE (16)
INDICES=(0, 0, 0, 0, 0)
AXX=(0.000000000000000E+00, 0)
AXY=(0.000000000000000E+00, 0)
AXZ=(0.000000000000000E+00, 0)
AYY=(0.000000000000000E+00, 0)
AYZ=(0.000000000000000E+00, 0)
AZZ=(0.000000000000000E+00, 0)
AX=(-0.592838567020995E-01, 0)
AY=(0.225536198486027E+00, 0)
AZ=(-0.972429353478697E+00, 0)
A0=(4.222012481265670E-06, 0)
0000000000000000000000000000000000000000000000
SURFACE (17)
INDICES=(0, 0, 0, 0, 0)
AXX=(0.000000000000000E+00, 0)
AXY=(0.000000000000000E+00, 0)
AXZ=(0.000000000000000E+00, 0)
AYY=(0.000000000000000E+00, 0)
AYZ=(0.000000000000000E+00, 0)
AZZ=(0.000000000000000E+00, 0)
AX=(-0.193559781656466E+00, 0)
AY=(0.493976576298720E-05, 0)
AZ=(-0.981088482707192E+00, 0)
A0=(4.200915240447980E-06, 0)
0000000000000000000000000000000000000000000000

SURFACE (18)
INDICES=(0, 0, 0, 0, 0)
AXX=(0.000000000000000E+00, 0)
AXY=(0.000000000000000E+00, 0)
AXZ=(0.000000000000000E+00, 0)
AYY=(0.000000000000000E+00, 0)
AYZ=(0.000000000000000E+00, 0)
AZZ=(0.000000000000000E+00, 0)
AX=(-0.592833369868559E-01, 0)
AY=(-0.225534221310335E+00, 0)
AZ=(-0.972429843728402E+00, 0)
A0=(4.222011833478610E-06, 0)
00000000000000000000000000000000000000000000000000
SURFACE (19)
INDICES=(0, 0, 0, 0, 0)
AXX=(0.000000000000000E+00, 0)
AXY=(0.000000000000000E+00, 0)
AXZ=(0.000000000000000E+00, 0)
AYY=(0.000000000000000E+00, 0)
AYZ=(0.000000000000000E+00, 0)
AZZ=(0.000000000000000E+00, 0)
AX=(0.156060836802792E+00, 0)
AY=(-0.140154446780401E+00, 0)
AZ=(-0.977753417925037E+00, 0)
A0=(4.209392845810320E-06, 0)
00000000000000000000000000000000000000000000000000
SURFACE (20)
INDICES=(0, 0, 0, 0, 0)
AXX=(0.000000000000000E+00, 0)
AXY=(0.000000000000000E+00, 0)
AXZ=(0.000000000000000E+00, 0)
AYY=(0.000000000000000E+00, 0)
AYZ=(0.000000000000000E+00, 0)
AZZ=(0.000000000000000E+00, 0)
AX=(0.156060944481120E+00, 0)
AY=(0.140149620425506E+00, 0)
AZ=(-0.977754092552032E+00, 0)
A0=(4.209391822847660E-06, 0)
00000000000000000000000000000000000000000000000000
SURFACE (21)
INDICES=(0, 0, 0, 0, 0)
AXX=(0.000000000000000E+00, 0)
AXY=(0.000000000000000E+00, 0)
AXZ=(0.000000000000000E+00, 0)
AYY=(0.000000000000000E+00, 0)
AYZ=(0.000000000000000E+00, 0)
AZZ=(0.000000000000000E+00, 0)
AX=(0.818202874588690E-01, 0)
AY=(0.311263521582366E+00, 0)
AZ=(0.946794835585985E+00, 0)
A0=(0.473397417792993E-05, 0)
00000000000000000000000000000000000000000000000000
SURFACE (22)
INDICES=(0, 0, 0, 0, 0)
AXX=(0.000000000000000E+00, 0)
AXY=(0.000000000000000E+00, 0)

AXZ=(0.000000000000000E+00, 0)
AYY=(0.000000000000000E+00, 0)
AYZ=(0.000000000000000E+00, 0)
AZZ=(0.000000000000000E+00, 0)
AX=(-0.216457555128471E+00, 0)
AY=(0.194396808066742E+00, 0)
AZ=(0.956742393667840E+00, 0)
A0=(0.478371196833920E-05, 0)
0000000000000000000000000000000000000000000000
SURFACE (23)
INDICES=(0, 0, 0, 0, 0)
AXX=(0.000000000000000E+00, 0)
AXY=(0.000000000000000E+00, 0)
AXZ=(0.000000000000000E+00, 0)
AYY=(0.000000000000000E+00, 0)
AYZ=(0.000000000000000E+00, 0)
AZZ=(0.000000000000000E+00, 0)
AX=(-0.216457822951077E+00, 0)
AY=(-0.194390683684687E+00, 0)
AZ=(0.956743577443758E+00, 0)
A0=(0.478371788721879E-05, 0)
0000000000000000000000000000000000000000000000
SURFACE (24)
INDICES=(0, 0, 0, 0, 0)
AXX=(0.000000000000000E+00, 0)
AXY=(0.000000000000000E+00, 0)
AXZ=(0.000000000000000E+00, 0)
AYY=(0.000000000000000E+00, 0)
AYZ=(0.000000000000000E+00, 0)
AZZ=(0.000000000000000E+00, 0)
AX=(0.818209213009259E-01, 0)
AY=(-0.311265932865886E+00, 0)
AZ=(0.946793988085369E+00, 0)
A0=(0.473396994042685E-05, 0)
0000000000000000000000000000000000000000000000
SURFACE (25)
INDICES=(0, 0, 0, 0, 0)
AXX=(0.000000000000000E+00, 0)
AXY=(0.000000000000000E+00, 0)
AXZ=(0.000000000000000E+00, 0)
AYY=(0.000000000000000E+00, 0)
AYZ=(0.000000000000000E+00, 0)
AZZ=(0.000000000000000E+00, 0)
AX=(0.269320732215448E+00, 0)
AY=(-0.640690675171039E-05, 0)
AZ=(0.963050540292609E+00, 0)
A0=(0.481525270146305E-05, 0)
0000000000000000000000000000000000000000000000
SURFACE (26)
INDICES=(0, 0, 0, 0, 0)
AXX=(0.000000000000000E+00, 0)
AXY=(0.000000000000000E+00, 0)
AXZ=(0.000000000000000E+00, 0)
AYY=(0.000000000000000E+00, 0)
AYZ=(0.000000000000000E+00, 0)
AZZ=(0.000000000000000E+00, 0)

```
     AX=( 0.253275833132285E+00,  0)
     AY=( 0.963520542409402E+00,  0)
     AZ=( 0.864841991709329E-01,  0)
     A0=( 0.327675332441755E-05,  0)
000000000000000000000000000000000000000000000
SURFACE ( 27)
INDICES=( 0, 0, 0, 0, 0)
    AXX=( 0.000000000000000E+00,  0)
    AXY=( 0.000000000000000E+00,  0)
    AXZ=( 0.000000000000000E+00,  0)
    AYY=( 0.000000000000000E+00,  0)
    AYZ=( 0.000000000000000E+00,  0)
    AZZ=( 0.000000000000000E+00,  0)
     AX=(-0.994478958192294E+00,  0)
     AY=( 0.236577923253873E-04,  0)
     AZ=(-0.104936176569753E+00,  0)
     A0=( 0.397585228893093E-05,  0)
000000000000000000000000000000000000000000000
SURFACE ( 28)
INDICES=( 0, 0, 0, 0, 0)
    AXX=( 0.000000000000000E+00,  0)
    AXY=( 0.000000000000000E+00,  0)
    AXZ=( 0.000000000000000E+00,  0)
    AYY=( 0.000000000000000E+00,  0)
    AYZ=( 0.000000000000000E+00,  0)
    AZZ=( 0.000000000000000E+00,  0)
     AX=(-0.740524792584101E+00,  0)
     AY=( 0.665052582189591E+00,  0)
     AZ=( 0.965820609183231E-01,  0)
     A0=( 0.365944782689502E-05,  0)
000000000000000000000000000000000000000000000
SURFACE ( 29)
INDICES=( 0, 0, 0, 0, 0)
    AXX=( 0.000000000000000E+00,  0)
    AXY=( 0.000000000000000E+00,  0)
    AXZ=( 0.000000000000000E+00,  0)
    AYY=( 0.000000000000000E+00,  0)
    AYZ=( 0.000000000000000E+00,  0)
    AZZ=( 0.000000000000000E+00,  0)
     AX=(-0.253275833132285E+00,  0)
     AY=(-0.963520542409402E+00,  0)
     AZ=(-0.864841991709329E-01,  0)
     A0=( 0.327675332441755E-05,  0)
000000000000000000000000000000000000000000000
SURFACE ( 30)
INDICES=( 0, 0, 0, 0, 0)
    AXX=( 0.000000000000000E+00,  0)
    AXY=( 0.000000000000000E+00,  0)
    AXZ=( 0.000000000000000E+00,  0)
    AYY=( 0.000000000000000E+00,  0)
    AYZ=( 0.000000000000000E+00,  0)
    AZZ=( 0.000000000000000E+00,  0)
     AX=(-0.740535263679589E+00,  0)
     AY=(-0.665040210775062E+00,  0)
     AZ=( 0.965869623668857E-01,  0)
     A0=( 0.365951371493133E-05,  0)
```

000000000000000000000000000000000000000000000000
SURFACE (31)
INDICES=(0, 0, 0, 0, 0)
AXX=(0.000000000000000E+00, 0)
AXY=(0.000000000000000E+00, 0)
AXZ=(0.000000000000000E+00, 0)
AYY=(0.000000000000000E+00, 0)
AYZ=(0.000000000000000E+00, 0)
AZZ=(0.000000000000000E+00, 0)
AX=(0.740524792584101E+00, 0)
AY=(-0.665052582189591E+00, 0)
AZ=(-0.965820609183231E-01, 0)
A0=(0.365944782689502E-05, 0)
00000000000000000000000000000000000000000000000
SURFACE (32)
INDICES=(0, 0, 0, 0, 0)
AXX=(0.000000000000000E+00, 0)
AXY=(0.000000000000000E+00, 0)
AXZ=(0.000000000000000E+00, 0)
AYY=(0.000000000000000E+00, 0)
AYZ=(0.000000000000000E+00, 0)
AZZ=(0.000000000000000E+00, 0)
AX=(0.253275833132285E+00, 0)
AY=(-0.963520542409402E+00, 0)
AZ=(0.864841991709329E-01, 0)
A0=(0.327672799683424E-05, 0)
00000000000000000000000000000000000000000000000
SURFACE (33)
INDICES=(0, 0, 0, 0, 0)
AXX=(0.000000000000000E+00, 0)
AXY=(0.000000000000000E+00, 0)
AXZ=(0.000000000000000E+00, 0)
AYY=(0.000000000000000E+00, 0)
AYZ=(0.000000000000000E+00, 0)
AZZ=(0.000000000000000E+00, 0)
AX=(0.740535263679589E+00, 0)
AY=(0.665040210775062E+00, 0)
AZ=(-0.965869623668857E-01, 0)
A0=(0.365951371493133E-05, 0)
00000000000000000000000000000000000000000000000
SURFACE (34)
INDICES=(0, 0, 0, 0, 0)
AXX=(0.000000000000000E+00, 0)
AXY=(0.000000000000000E+00, 0)
AXZ=(0.000000000000000E+00, 0)
AYY=(0.000000000000000E+00, 0)
AYZ=(0.000000000000000E+00, 0)
AZZ=(0.000000000000000E+00, 0)
AX=(0.994478958192294E+00, 0)
AY=(-0.236577923253873E-04, 0)
AZ=(0.104936176569753E+00, 0)
A0=(0.397585228893093E-05, 0)
00000000000000000000000000000000000000000000000
SURFACE (35)
INDICES=(0, 0, 0, 0, 0)
AXX=(0.000000000000000E+00, 0)

AXY=(0.000000000000000E+00, 0)
AXZ=(0.000000000000000E+00, 0)
AYY=(0.000000000000000E+00, 0)
AYZ=(0.000000000000000E+00, 0)
AZZ=(0.000000000000000E+00, 0)
AX=(-0.253275833132285E+00, 0)
AY=(0.963520542409402E+00, 0)
AZ=(-0.864841991709329E-01, 0)
A0=(0.327672799683424E-05, 0)
0000000000000000000000000000000000000000000000000000000000000000
SURFACE (36)
INDICES=(0, 0, 0, 0, 0)
AXX=(0.000000000000000E+00, 0)
AXY=(0.000000000000000E+00, 0)
AXZ=(0.000000000000000E+00, 0)
AYY=(0.000000000000000E+00, 0)
AYZ=(0.000000000000000E+00, 0)
AZZ=(0.000000000000000E+00, 0)
AX=(-0.818209213009259E-01, 0)
AY=(0.311265932865886E+00, 0)
AZ=(-0.946793988085369E+00, 0)
A0=(0.473396994042685E-05, 0)
0000000000000000000000000000000000000000000000000000000000000000
SURFACE (37)
INDICES=(0, 0, 0, 0, 0)
AXX=(0.000000000000000E+00, 0)
AXY=(0.000000000000000E+00, 0)
AXZ=(0.000000000000000E+00, 0)
AYY=(0.000000000000000E+00, 0)
AYZ=(0.000000000000000E+00, 0)
AZZ=(0.000000000000000E+00, 0)
AX=(-0.269320732215448E+00, 0)
AY=(0.640690675171039E-05, 0)
AZ=(-0.963050540292609E+00, 0)
A0=(0.481525270146305E-05, 0)
0000000000000000000000000000000000000000000000000000000000000000
SURFACE (38)
INDICES=(0, 0, 0, 0, 0)
AXX=(0.000000000000000E+00, 0)
AXY=(0.000000000000000E+00, 0)
AXZ=(0.000000000000000E+00, 0)
AYY=(0.000000000000000E+00, 0)
AYZ=(0.000000000000000E+00, 0)
AZZ=(0.000000000000000E+00, 0)
AX=(-0.818202874588690E-01, 0)
AY=(-0.311263521582366E+00, 0)
AZ=(-0.946794835585985E+00, 0)
A0=(0.473397417792993E-05, 0)
0000000000000000000000000000000000000000000000000000000000000000
SURFACE (39)
INDICES=(0, 0, 0, 0, 0)
AXX=(0.000000000000000E+00, 0)
AXY=(0.000000000000000E+00, 0)
AXZ=(0.000000000000000E+00, 0)
AYY=(0.000000000000000E+00, 0)
AYZ=(0.000000000000000E+00, 0)

```
    AZZ=( 0.000000000000000E+00,  0)
     AX=( 0.216457555128471E+00,  0)
     AY=(-0.194396808066742E+00,  0)
     AZ=(-0.956742393667840E+00,  0)
     A0=( 0.478371196833920E-05,  0)
00000000000000000000000000000000000000000000000000
SURFACE ( 40)
INDICES=( 0, 0, 0, 0, 0)
    AXX=( 0.000000000000000E+00,  0)
    AXY=( 0.000000000000000E+00,  0)
    AXZ=( 0.000000000000000E+00,  0)
    AYY=( 0.000000000000000E+00,  0)
    AYZ=( 0.000000000000000E+00,  0)
    AZZ=( 0.000000000000000E+00,  0)
     AX=( 0.216457822951077E+00,  0)
     AY=( 0.194390683684687E+00,  0)
     AZ=(-0.956743577443758E+00,  0)
     A0=( 0.478371788721879E-05,  0)
00000000000000000000000000000000000000000000000000
SURFACE ( 41)
INDICES=( 0, 0, 0, 1,-1)
Z-SCALE=( 3.500000000000000E-06,  0)
0000000000000000000000000000000000000000000000000
SURFACE ( 42)
INDICES=( 0, 0, 0, 1, 1)
Z-SCALE=( 3.500000000000000E-06,  0)
0000000000000000000000000000000000000000000000000
SURFACE ( 43)
INDICES=( 1, 1, 0, 0,-1)
X-SCALE=( 9.528202108382620E-08,  0)
Y-SCALE=( 7.940168423652180E-08,  0)
0000000000000000000000000000000000000000000000000
SURFACE ( 44)
INDICES=( 1, 1, 0, 0,-1)
X-SCALE=( 3.286335345031000E-07,  0)
Y-SCALE=( 2.738612787525830E-07,  0)
0000000000000000000000000000000000000000000000000
SURFACE ( 45)
INDICES=( 1, 1, 0, 0,-1)
X-SCALE=( 4.239155555869260E-07,  0)
Y-SCALE=( 3.532629629891050E-07,  0)
0000000000000000000000000000000000000000000000000
SURFACE ( 46)
INDICES=( 1, 1, 0, 0,-1)
X-SCALE=( 6.572670690061990E-07,  0)
Y-SCALE=( 5.477225575051660E-07,  0)
0000000000000000000000000000000000000000000000000
SURFACE ( 47)
INDICES=( 1, 1, 0, 0,-1)
X-SCALE=( 7.525490900900260E-07,  0)
Y-SCALE=( 6.271242417416880E-07,  0)
0000000000000000000000000000000000000000000000000
SURFACE ( 48)
INDICES=( 1, 1, 0, 0,-1)
X-SCALE=( 9.859006035092990E-07,  0)
Y-SCALE=( 8.215838362577490E-07,  0)
```

0000000000000000000000000000000000000000000000000
SURFACE (49)
INDICES=(1, 1, 0, 0,-1)
X-SCALE=(1.081182624593130E-06, 0)
Y-SCALE=(9.009855204942710E-07, 0)
0000000000000000000000000000000000000000000000000
SURFACE (50)
INDICES=(1, 1, 0, 0,-1)
X-SCALE=(1.314534138012400E-06, 0)
Y-SCALE=(1.095445115010330E-06, 0)
0000000000000000000000000000000000000000000000000
SURFACE (51)
INDICES=(1, 1, 0, 0,-1)
X-SCALE=(1.409816159096220E-06, 0)
Y-SCALE=(1.174846799246850E-06, 0)
0000000000000000000000000000000000000000000000000
SURFACE (52)
INDICES=(1, 1, 0, 0,-1)
X-SCALE=(1.643167672515500E-06, 0)
Y-SCALE=(1.369306393762920E-06, 0)
0000000000000000000000000000000000000000000000000
SURFACE (53)
INDICES=(1, 1, 0, 0,-1)
X-SCALE=(1.738449693599320E-06, 0)
Y-SCALE=(1.448708077999440E-06, 0)
0000000000000000000000000000000000000000000000000
SURFACE (54)
INDICES=(1, 1, 0, 0,-1)
X-SCALE=(1.971801207018600E-06, 0)
Y-SCALE=(1.643167672515500E-06, 0)
0000000000000000000000000000000000000000000000000
SURFACE (55)
INDICES=(1, 1, 0, 0,-1)
X-SCALE=(2.067083228102420E-06, 0)
Y-SCALE=(1.722569356752020E-06, 0)
0000000000000000000000000000000000000000000000000
SURFACE (56)
INDICES=(1, 1, 0, 0,-1)
X-SCALE=(2.300434741521700E-06, 0)
Y-SCALE=(1.917028951268080E-06, 0)
0000000000000000000000000000000000000000000000000
SURFACE (57)
INDICES=(1, 1, 0, 0,-1)
X-SCALE=(2.395716762605520E-06, 0)
Y-SCALE=(1.996430635504600E-06, 0)
0000000000000000000000000000000000000000000000000
SURFACE (58)
INDICES=(1, 1, 0, 0,-1)
X-SCALE=(2.629068276024800E-06, 0)
Y-SCALE=(2.190890230020670E-06, 0)
0000000000000000000000000000000000000000000000000
SURFACE (59)
INDICES=(1, 1, 0, 0,-1)
X-SCALE=(3.400000000000000E-06, 0)
Y-SCALE=(2.833333333333330E-06, 0)
0000000000000000000000000000000000000000000000000

```
SURFACE ( 60)
INDICES=( 1, 1, 0, 0,-1)
X-SCALE=( 3.600000000000000E-06,  0)
Y-SCALE=( 3.000000000000000E-06,  0)
00000000000000000000000000000000000000000000000000
SURFACE ( 61)    PLANE Z=1000.0
INDICES=( 0, 0, 0, 1,-1)
Z-SCALE=( 1.000000000000000E-05,  0)
000000000000000000000000000000000000000000000000000
SURFACE ( 62)    PLANE Z=-1000.0
INDICES=( 0, 0, 0, 1, 1)
Z-SCALE=( 1.000000000000000E-05,  0)
00000000000000000000000000000000000000000000000000
SURFACE ( 63)    PLANE X=1000.0
INDICES=( 0, 0, 0, 0, 0)
     AX=( 1.000000000000000E+00,  0)
     A0=(-2.000000000000000E-05,  0)
000000000000000000000000000000000000000000000000000
SURFACE ( 64)    PLANE X=-1000.0
INDICES=( 0, 0, 0, 0, 0)
     AX=( 1.000000000000000E+00,  0)
     A0=( 2.000000000000000E-05,  0)
00000000000000000000000000000000000000000000000000
BODY    (  1)
MATERIAL(  3)
SURFACE ( 41), SIDE POINTER=(-1)
SURFACE ( 42), SIDE POINTER=( 1)
SURFACE ( 43), SIDE POINTER=( 1)
SURFACE ( 44), SIDE POINTER=(-1)
00000000000000000000000000000000000000000000000000
BODY    (  2)
MATERIAL(  3)
SURFACE ( 41), SIDE POINTER=(-1)
SURFACE ( 42), SIDE POINTER=( 1)
SURFACE ( 45), SIDE POINTER=( 1)
SURFACE ( 46), SIDE POINTER=(-1)
00000000000000000000000000000000000000000000000000
BODY    (  3)
MATERIAL(  3)
SURFACE ( 41), SIDE POINTER=(-1)
SURFACE ( 42), SIDE POINTER=( 1)
SURFACE ( 47), SIDE POINTER=( 1)
SURFACE ( 48), SIDE POINTER=(-1)
00000000000000000000000000000000000000000000000000
BODY    (  4)
MATERIAL(  3)
SURFACE ( 41), SIDE POINTER=(-1)
SURFACE ( 42), SIDE POINTER=( 1)
SURFACE ( 49), SIDE POINTER=( 1)
SURFACE ( 50), SIDE POINTER=(-1)
00000000000000000000000000000000000000000000000000
BODY    (  5)
MATERIAL(  3)
SURFACE ( 41), SIDE POINTER=(-1)
SURFACE ( 42), SIDE POINTER=( 1)
SURFACE ( 51), SIDE POINTER=( 1)
```

SURFACE (52), SIDE POINTER=(-1)
000000000000000000000000000000000000000000000
BODY (6)
MATERIAL(3)
SURFACE (41), SIDE POINTER=(-1)
SURFACE (42), SIDE POINTER=(1)
SURFACE (53), SIDE POINTER=(1)
SURFACE (54), SIDE POINTER=(-1)
000000000000000000000000000000000000000000000
BODY (7)
MATERIAL(3)
SURFACE (41), SIDE POINTER=(-1)
SURFACE (42), SIDE POINTER=(1)
SURFACE (55), SIDE POINTER=(1)
SURFACE (56), SIDE POINTER=(-1)
000000000000000000000000000000000000000000000
BODY (8)
MATERIAL(3)
SURFACE (41), SIDE POINTER=(-1)
SURFACE (42), SIDE POINTER=(1)
SURFACE (57), SIDE POINTER=(1)
SURFACE (58), SIDE POINTER=(-1)
000000000000000000000000000000000000000000000
MODULE (9) INNER CAPSID
MATERIAL(1)
BODY (1)
BODY (2)
BODY (3)
BODY (4)
BODY (5)
BODY (6)
BODY (7)
BODY (8)
SURFACE (1), SIDE POINTER=(1)
SURFACE (2), SIDE POINTER=(1)
SURFACE (3), SIDE POINTER=(1)
SURFACE (4), SIDE POINTER=(1)
SURFACE (5), SIDE POINTER=(1)
SURFACE (6), SIDE POINTER=(1)
SURFACE (7), SIDE POINTER=(1)
SURFACE (8), SIDE POINTER=(1)
SURFACE (9), SIDE POINTER=(1)
SURFACE (10), SIDE POINTER=(1)
SURFACE (11), SIDE POINTER=(1)
SURFACE (12), SIDE POINTER=(1)
SURFACE (13), SIDE POINTER=(1)
SURFACE (14), SIDE POINTER=(1)
SURFACE (15), SIDE POINTER=(1)
SURFACE (16), SIDE POINTER=(1)
SURFACE (17), SIDE POINTER=(1)
SURFACE (18), SIDE POINTER=(1)
SURFACE (19), SIDE POINTER=(1)
SURFACE (20), SIDE POINTER=(1)
0000000000000000000000000000000000000000000000000000000000
MODULE (10) OUTER CAPSID
MATERIAL(2)

```
MODULE  (  9)
SURFACE ( 21), SIDE POINTER=( 1)
SURFACE ( 22), SIDE POINTER=( 1)
SURFACE ( 23), SIDE POINTER=( 1)
SURFACE ( 24), SIDE POINTER=( 1)
SURFACE ( 25), SIDE POINTER=( 1)
SURFACE ( 26), SIDE POINTER=( 1)
SURFACE ( 27), SIDE POINTER=( 1)
SURFACE ( 28), SIDE POINTER=( 1)
SURFACE ( 29), SIDE POINTER=( 1)
SURFACE ( 30), SIDE POINTER=( 1)
SURFACE ( 31), SIDE POINTER=( 1)
SURFACE ( 32), SIDE POINTER=( 1)
SURFACE ( 33), SIDE POINTER=( 1)
SURFACE ( 34), SIDE POINTER=( 1)
SURFACE ( 35), SIDE POINTER=( 1)
SURFACE ( 36), SIDE POINTER=( 1)
SURFACE ( 37), SIDE POINTER=( 1)
SURFACE ( 38), SIDE POINTER=( 1)
SURFACE ( 39), SIDE POINTER=( 1)
SURFACE ( 40), SIDE POINTER=( 1)
00000000000000000000000000000000000000000000000000
Z-SHIFT=( 0.000000000000000E-00,  0)
00000000000000000000000000000000000000000000000000
MODULE  ( 11)
MATERIAL(  1)
SURFACE ( 61), SIDE POINTER=(-1)
SURFACE ( 62), SIDE POINTER=( 1)
SURFACE ( 63), SIDE POINTER=(-1)
SURFACE ( 64), SIDE POINTER=( 1)
MODULE  ( 10)
00000000000000000000000000000000000000000000000000
END      00000000000000000000000000000000000000000
```

For Orientation Shown in Fig 9c replace last 9 lines by

```
111111111111111111111111111111111111111111111111111111111111111111111
  THETA=( 9.000000000000000E+01,  0)
Z-SHIFT=( 0.000000000000000E-00,  0)
00000000000000000000000000000000000000000000000000
MODULE  ( 11)
MATERIAL(  1)
SURFACE ( 61), SIDE POINTER=(-1)
SURFACE ( 62), SIDE POINTER=( 1)
SURFACE ( 63), SIDE POINTER=(-1)
SURFACE ( 64), SIDE POINTER=( 1)
MODULE  ( 10)
111111111111111111111111111111111111111111111111111111111111111111111
  OMEGA=( 9.000000000000000E+01,  0)
  THETA=( 1.000000000000000E+01,  0)
    PHI=(-9.000000000000000E+01,  0)
00000000000000000000000000000000000000000000000000
END      00000000000000000000000000000000000000000
```

To change the depth set Z-SHIFT to appropriate distance in cm, +ve for lower half , negative for upper half.
